# 3D Printing of Continuous-Fiber-Reinforced Composites: Advances in Multifunctional Integration and Intelligent Manufacturing

**DOI:** 10.3390/polym18182285

**Published:** 2026-09-19

**Authors:** Shuo Han, Yu Long, Ming Cai, Qihua Ma, Baozhong Sun, Geoffrey I. N. Waterhouse

**Affiliations:** 1 School of Air Transportation, Shanghai University of Engineering Science, Shanghai 201620, China; 15966882787@163.com (S.H.);; 2 Yongjiang Laboratory, Ningbo 315202, China; 3 Shanghai Frontiers Science Center of Advanced Textiles, College of Textiles, Donghua University, Shanghai 201620, China; 4 School of Chemical Sciences, The University of Auckland, Auckland 1142, New Zealand

**Keywords:** continuous-fiber-reinforced composites, additive manufacturing, smart architecture, multifunctional composites, intelligent manufacturing

## Abstract

Extrusion-based additive manufacturing has emerged as a transformative route for 3D-printed continuous-fiber-reinforced polymer composites (3DP-CFRPCs), offering unprecedented opportunities to engineer lightweight structures with programmable mechanical and multifunctional properties. However, existing studies largely treat constituent materials, printing processes, structural design, and functional integration as isolated research topics, limiting the development of robust design principles for intelligent composite systems. This review proposes an architecture-centric smart architecture framework that extends conventional material–process–structure–property relationships by explicitly incorporating reinforcement-path architecture, interface/defect evolution, multifunctional states, and cyber–physical feedback as coupled design and state variables. Within this framework, we critically synthesize the coupled roles of matrix rheology, fiber impregnation, interfacial bonding, and fiber trajectory engineering in determining printability, defect evolution, and mechanical performance. Recent advances in co-extrusion technologies, multi-axis printing, topology optimization, and programmable fiber placement are further discussed as enabling strategies for architected composite design. Particular emphasis is placed on defect-controlled reliability, multifunctional integration, and emerging AI-enabled digital twins that facilitate closed-loop process optimization and intelligent manufacturing. Finally, the remaining challenges regarding scalability, repeatability, and intelligent composite architectures are outlined to accelerate the transition from laboratory-scale demonstrations to industrial deployment.

## 1. Introduction

The increasing demand for lightweight, high-strength, and multifunctional structures in aerospace, transportation, energy, robotics, and biomedical engineering fields is accelerating the convergence of advanced composite materials and digital manufacturing technologies [1,2,3]. As modern engineering systems evolve toward greater structural efficiency, functional integration, and operational intelligence, conventional manufacturing approaches are increasingly constrained by geometric complexity, material waste, moldless manufacturing, and limited design flexibility [4,5]. Additive manufacturing (AM) has emerged as a transformative manufacturing paradigm capable of fabricating highly customized components directly from digital models, enabling unprecedented freedom in structural design while reducing tooling requirements and material consumption [6,7]. However, the current state of 3DP-CFRPCs is still fragmented, with material selection, process control, structural design, and functional integration often treated as separate problems rather than as interdependent design variables [8,9,10,11,12,13,14].

Among various AM technologies, fused deposition modeling (FDM) of 3DP-CFRPCs has drawn considerable interest. This is primarily because it synergistically combines complex structural design integration with the outstanding specific stiffness and strength of continuous-fiber-reinforced composites [15,16,17]. Unlike short fiber-reinforced composites, continuous fibers provide efficient load transfer pathways and enable the fabrication of lightweight structures with mechanical properties approaching those of conventionally autoclave-consolidated composites [18,19].

Despite these advances, the performance and reliability of 3DP-CFRPCs remain strongly limited by the intrinsic coupling among material behavior, process physics, structural architecture, and functional requirements. Process-induced defects such as insufficient impregnation, void formation, fiber waviness, interlayer discontinuities, and interface degradation continue to restrict the attainable fiber-volume fraction and interlaminar performance [20,21,22,23,24,25,26,27]. At the same time, the increasing demand for complex geometries and multifunctional capabilities introduces additional challenges regarding topology optimization, fiber-path planning, manufacturability constraints, and process control [28]. Consequently, the design of continuous-fiber-reinforced composites can no longer be treated as a sequential workflow in which materials, manufacturing, structural design, and functional integration are optimized independently. Instead, these factors must be considered as forming a tightly coupled system spanning multiple length scales and disciplinary boundaries [29,30].

In parallel, emerging developments in artificial intelligence, digital twins, in situ sensing, and closed-loop manufacturing are reshaping the future of composite fabrication. Real-time monitoring, machine-learning-assisted process optimization, and adaptive control strategies are enabling a transition from experience-driven manufacturing toward data-centric and autonomous production systems [31,32]. Furthermore, multifunctional concepts such as self-sensing, self-healing, thermal management, shape-memory actuation, and embedded electronics are transforming continuous-fiber-reinforced composites from passive load-bearing materials into intelligent structural systems capable of environmental perception, damage response, and functional adaptation. These advances indicate that the future of 3DP-CFRPCs lies not only in improving mechanical performance, but also in achieving the seamless integration of structure, function, and intelligence [33,34].

**Figure 1:**
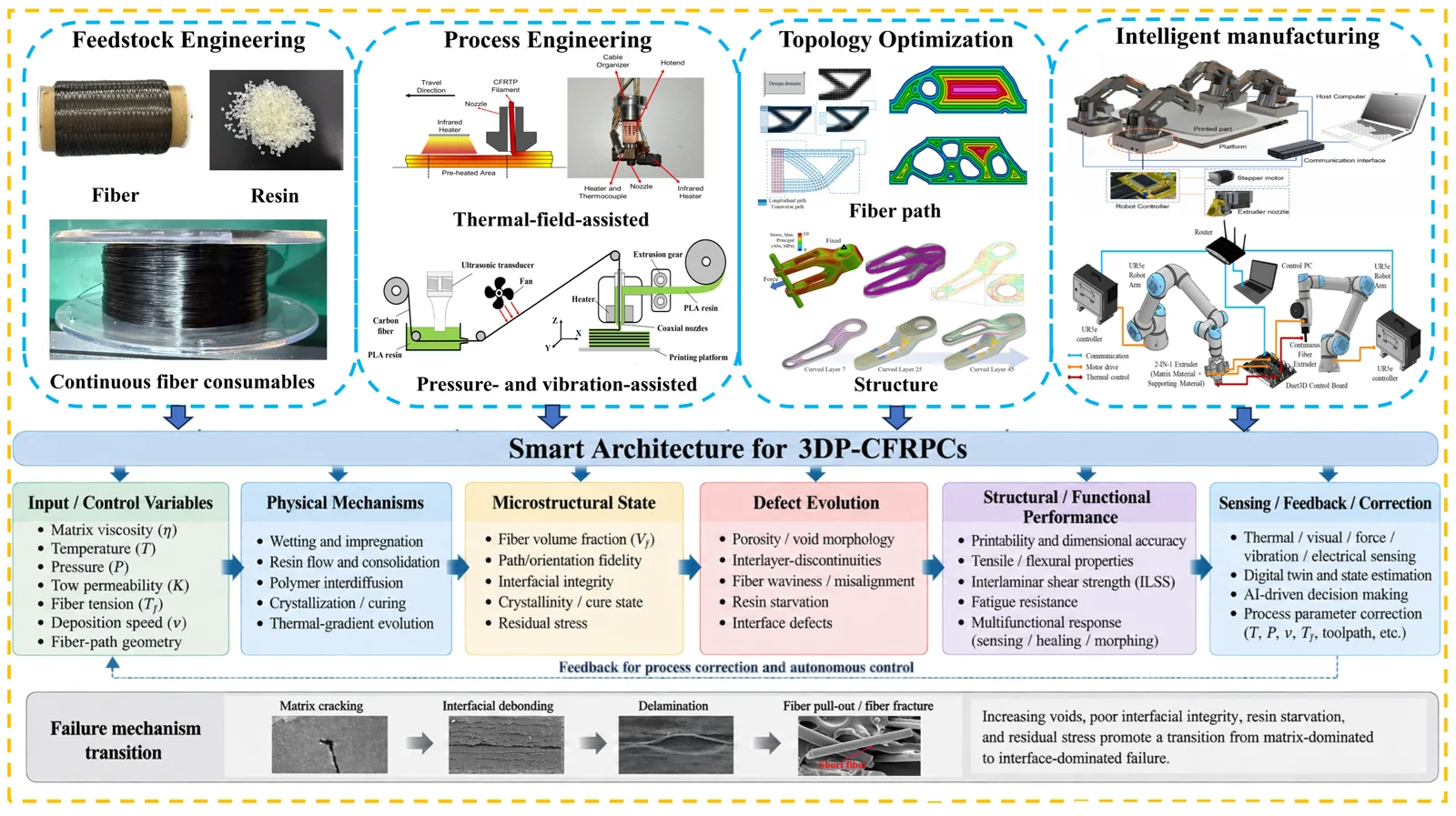
Smart architecture framework for 3DP-CFRPCs expressed as a closed-loop process–state–architecture–performance–control map [35,36,37,38,39,40,41,42,43,44,45,46,47,48].

Recent studies and reviews have increasingly integrated materials, processing, structural design, functionality, and intelligent manufacturing into continuous-fiber additive manufacturing. The remaining need is therefore not simply a broader collection of these topics, but a framework that makes their physical couplings and measurable intermediate states explicit. Building on established material–process–structure–property relationships, smart architecture is proposed here as an architecture-centric and experimentally tractable framework for 3DP-CFRPCs. Its distinctive feature is the explicit treatment of fiber orientation, trajectory, curvature, continuity, and topology as design and manufacturing state variables; impregnation, interfacial integrity, porosity, fiber waviness, crystallinity, and residual stress as evolving intermediate states; and sensing, digital twins, and AI-enabled control as feedback mechanisms. As illustrated in Figure 1, controllable variables such as matrix viscosity, temperature, pressure, tow permeability, fiber tension, deposition speed, and path geometry govern impregnation, consolidation, polymer interdiffusion, and thermal evolution, which subsequently determine microstructural state, defect development, structural reliability, and multifunctional performance. Smart architecture, therefore, extends a predominantly forward material–process–structure–property relationship toward a closed-loop process–state–architecture–performance–control framework. Through this lens, the present review critically synthesizes recent advances in 3DP-CFRPCs, identifies the key coupling mechanisms and measurable state variables governing their evolution, and highlights the unresolved challenges that must be addressed to achieve scalable, reliable, multifunctional, and ultimately autonomous composite manufacturing. 

As summarized in Table 1**,** recent reviews have already established comprehensive coverage of materials, processing, mechanical behavior, functional composites, non-planar manufacturing, process planning, and structural optimization. The contribution of the present review therefore does not arise from simply extending the breadth of topics covered. Instead, smart architecture is introduced as a relational framework that reorganizes these established research domains according to the physical variables and intermediate states through which they interact. Three distinctions are emphasized. First, fiber architecture—including orientation, trajectory, curvature, continuity, and topology—is treated as an explicit design and manufacturing state rather than only as a structural outcome. Second, impregnation quality, interfacial integrity, porosity, fiber waviness, and residual stress are treated as evolving intermediate states that transmit feedstock and process variability to structural reliability. Third, in situ sensing, digital twins, and AI-enabled control are incorporated as feedback mechanisms through which measured process and defect states can modify processing conditions and fiber-path execution. Smart architecture therefore extends the conventional forward material–process–structure–property relationship toward a bidirectionally coupled material–process–interface/defect–architecture–performance–control framework.

### Review Scope and Literature-Selection Methodology

This article is structured as a critical narrative review informed by a systematic literature-search procedure rather than as a PRISMA-type systematic review. Web of Science Core Collection and Scopus were used as the principal bibliographic databases, with Google Scholar employed for supplementary searches and backward/forward citation tracking. The search covered publications from 2018 to 2026 and combined the core terms (“continuous fiber” OR “continuous fibre”) AND (“3D printing” OR “additive manufacturing” OR “material extrusion” OR “fused filament fabrication”) with topic-specific terms including impregnation, consolidation, defect, fiber path, topology optimization, multi-axis printing, multifunctional composite, self-sensing, self-healing, digital twin, artificial intelligence, mechanical performance, and fatigue. English-language publications directly addressing continuous-fiber polymer additive manufacturing were prioritized. Related studies from the broader additive-manufacturing or composite-manufacturing literature were included only when they provided directly transferable mechanisms or methods relevant to the smart architecture framework. Peer-reviewed journal articles were prioritized; conference papers were included when they reported relevant emerging technologies or experimental evidence, whereas preprints were used selectively and not as the sole basis for major quantitative conclusions. Duplicate, inaccessible, and clearly out-of-scope studies were excluded.

## 2. Feedstock Engineering

Material systems dictate the intrinsic processability and multifunctional potential of 3DP-CFRPCs by governing rheological behavior, fiber–matrix compatibility, and interfacial interactions. Within the smart architecture framework, feedstock engineering provides the material foundation for subsequent process regulation, structural optimization, and functional integration.

### 2.1. Matrix Materials and Processability

As shown in Table 2, the matrix system governs the material behavior throughout extrusion-based 3DP-CFRPCs, from nozzle flow and tow impregnation to road consolidation and interlayer diffusion [52]. Within the smart architecture framework, the matrix is not simply a passive binding phase but rather the medium that controls viscosity, heat transfer, wetting kinetics, and solidification behavior within the print head and deposited roads. Therefore, matrix selection directly defines the practical processing window and strongly influences dimensional fidelity, void formation, and interlayer bonding quality [53].

Thermoplastics are particularly attractive for continuous-fiber 3D printing because they support melt processing, rapid layer-wise deposition, and post-processing recyclability. However, their relatively high melt viscosity and narrow thermal window often limit fiber-bundle infiltration and inter-road fusion, especially when high printing speeds or thick fiber tows are used [54]. As a result, thermoplastic systems require careful coordination among nozzle temperature, residence time, and deposition speed to maintain stable flow while preserving geometric accuracy. Thermosets, by contrast, usually provide lower initial viscosity and more favorable wetting behavior, which facilitates impregnation and enables higher fiber-volume-fraction printing [55]. Yet their curing kinetics introduce additional process constraints, since gelation must be synchronized with deposition and consolidation to avoid premature solidification, residual stress buildup, and incomplete bonding. From a smart architecture perspective, the key distinction between these matrix families lies not only in chemistry, but in how they respond to the coupled thermal, rheological, and kinetic demands of layer-wise fabrication [56,57].

From a processing-window perspective, temperature, residence time, and deposition speed should therefore be considered as coupled variables rather than as independently optimized parameters. For thermoplastic matrices, increasing processing temperature generally decreases melt viscosity and facilitates fiber wetting, resin redistribution, and polymer-chain mobility across adjacent deposited roads. However, sufficient thermal exposure must also be maintained for interlayer diffusion before cooling and crystallization progressively restrict molecular mobility. Consequently, an excessively low temperature or short effective residence time can lead to incomplete impregnation and weak interlayer bonding, whereas excessive temperature or prolonged thermal exposure may reduce shape stability and increase the risk of matrix degradation. Deposition speed directly interacts with these effects because it regulates the time available for impregnation, wetting, and interfacial consolidation during continuous material delivery.

For thermosetting matrices, the corresponding processing window is controlled by the competition between low-viscosity wetting and cure-induced solidification. A sufficiently low initial viscosity favors infiltration into the fiber bundle and promotes interfacial contact, whereas premature gelation can restrict resin redistribution and compromise both impregnation and interlayer consolidation. Conversely, excessively slow curing may reduce dimensional stability after deposition. Therefore, gelation and cure kinetics must be coordinated with processing temperature, residence time, and deposition rate. Taken together, these relationships indicate that a useful matrix-processing window is determined by the coupled evolution of viscosity, wetting/impregnation, interlayer diffusion, and crystallization or cure kinetics rather than by processing temperature alone. The representative processing ranges and dominant kinetic constraints for thermoplastic and thermosetting matrices are summarized in Table 2.

Accordingly, matrix selection should be treated as a process-defining variable rather than as a material choice alone. An appropriate matrix must provide a sufficiently broad rheological and kinetic window to accommodate impregnation, deposition, interfacial consolidation, and subsequent solidification while preserving geometric fidelity [58]. Emerging reactive systems, such as vitrimer-based or dual-cure matrices, may further broaden this processing space by combining re-processability with improved consolidation control. Nevertheless, their practical implementation remains governed by the same requirement: matrix-state evolution must remain compatible with the transient thermal and kinematic conditions imposed by continuous-fiber 3D printing [59].

**Table 2:** Comparison of matrix resin classes for 3DP-CFRPCs.

Resin Class	Thermoplastics	Thermosets
Representative polymers	PLA, ABS, PP, PA6, PEEK, PEI	Epoxy; vinyl ester; polyester
Typical processing condition	Melt processing; material-specific nozzle temperature	Low-viscosity deposition followed by or coupled with curing
Dominant rheological/kinetic constraint	Melt viscosity; wetting/impregnation; interlayer diffusion; cooling/crystallization	Initial viscosity; wetting/impregnation; gelation and cure kinetics
Key processing-window variables	Temperature; residence time; deposition speed; pressure	Temperature; residence time; deposition rate; cure schedule
Main advantages	Melt-processable; recyclable; good toughness; easy filament fabrication	Excellent fiber wet-out; high thermal/chemical stability; high fiber-volume fraction
Main limitations	High melt viscosity; incomplete impregnation; crystallization/warpage; thermal degradation risk	Cure-time control; premature gelation; shrinkage/residual stress; longer processing cycle
References	[53,54,55,56]	[49,54,55,56]

### 2.2. Continuous-Fiber Reinforcements and Functional Roles

Fiber selection introduces a second layer of process-related constraints because the fiber phase determines not only the mechanical ceiling of the printed composite, but also how the material can be deposited, consolidated, and functionally activated during printing [60]. In 3DP-CFRPCs, the reinforcement is not merely a structural filler; it acts as a load-bearing skeleton that must be fed, steered, impregnated, and stabilized within the constraints of the nozzle architecture and toolpath geometry. As a result, fiber choice has a direct impact on printability, defect tolerance, and multifunctional performance [61,62].

As shown in Table 3, carbon fibers are the most widely adopted reinforcement in 3DP-CFRPCs because they combine high stiffness, high specific strength, and intrinsic electrical conductivity. From a printing standpoint, these characteristics make them especially suitable for structures that require simultaneous mechanical reinforcement, Joule heating, and embedded sensing [63]. However, carbon fibers also require careful control of tension, path curvature, and interfacial wetting to avoid waviness, breakage, or incomplete impregnation during deposition. Glass fibers offer lower cost and good electrical insulation, making them attractive when dielectric isolation or impact resistance is prioritized [64]. Aramid fibers provide high toughness and damage tolerance, but their lower compressive performance and more challenging surface compatibility can complicate interfacial consolidation during printing [65]. Natural fibers, while increasingly explored for sustainability-oriented applications, are more sensitive to moisture uptake, thermal degradation, and property variability, which limits their use in high-temperature or high-precision extrusion processes [66].

From the perspective of the printing process, fiber-material behavior is strongly coupled to path continuity, orientation fidelity, and interfacial consolidation [67]. A fiber that is mechanically superior in isolation may still perform poorly if it cannot be stably transported through the nozzle, adequately wetted by the matrix, or precisely aligned along the intended deposition path [68]. Therefore, fiber selection must be considered together with matrix rheology and toolpath design. Only through this coupled material–process optimization can continuous-fiber printing fully exploit its unique advantages in structural reinforcement, electrical functionality, thermal conduction, and damage-tolerant architecture [69,70].

**Table 3:** Comparison of continuous fiber types.

Fiber Type	Carbon	Glass	Aramid	Ramie	Flax
Tensile Strength (MPa)	3000–7000	2000–4500	3600	400–600	500–900
Modulus (GPa)	200–900	70–90	70–130	30–60	50–70
Density (g/cm^3^)	1.8	2.5	1.4	1.5	1.5
Strain to Failure (%)	1.5–2.0	4–5	2–4	2–3	1.2–1.6
Key Advantages	Ultra-high specific stiffness/strength; embedded heating	Low cost; High fracture strain; Corrosion resistance	Excellent impact/fatigue resistance; high strength-to-weight ratio	Biodegradable; low cost; good specific properties	Sustainable; low density; good stiffness-to-weight ratio
Primary Limitations	High cost; brittle impact behavior; poor compressive strength	Lower specific stiffness; higher density	UV degradation; poor transverse stiffness; moisture uptake	Variable quality; hydrophilicity; low thermal stability	Variability; lower strength; moisture sensitivity
References	[61,63,65]	[64,66]	[67,69]	[62,68]	[70]

## 3. Process Engineering

Within the smart architecture framework, process engineering orchestrates the transformation of engineered feedstocks into high-performance, multifunctional structures by controlling impregnation behavior, thermorheological evolution, consolidation quality, and defect development during fabrication. The synergistic integration of optimized impregnation strategies, assisted forming technologies, and process-induced defect control establishes the essential processing–microstructure–property linkage that underpins both reliable structural performance and intelligent manufacturing of 3DP-CFRPCs.

### 3.1. Impregnation Strategies for Low-Defect Composites

**Figure 2:**
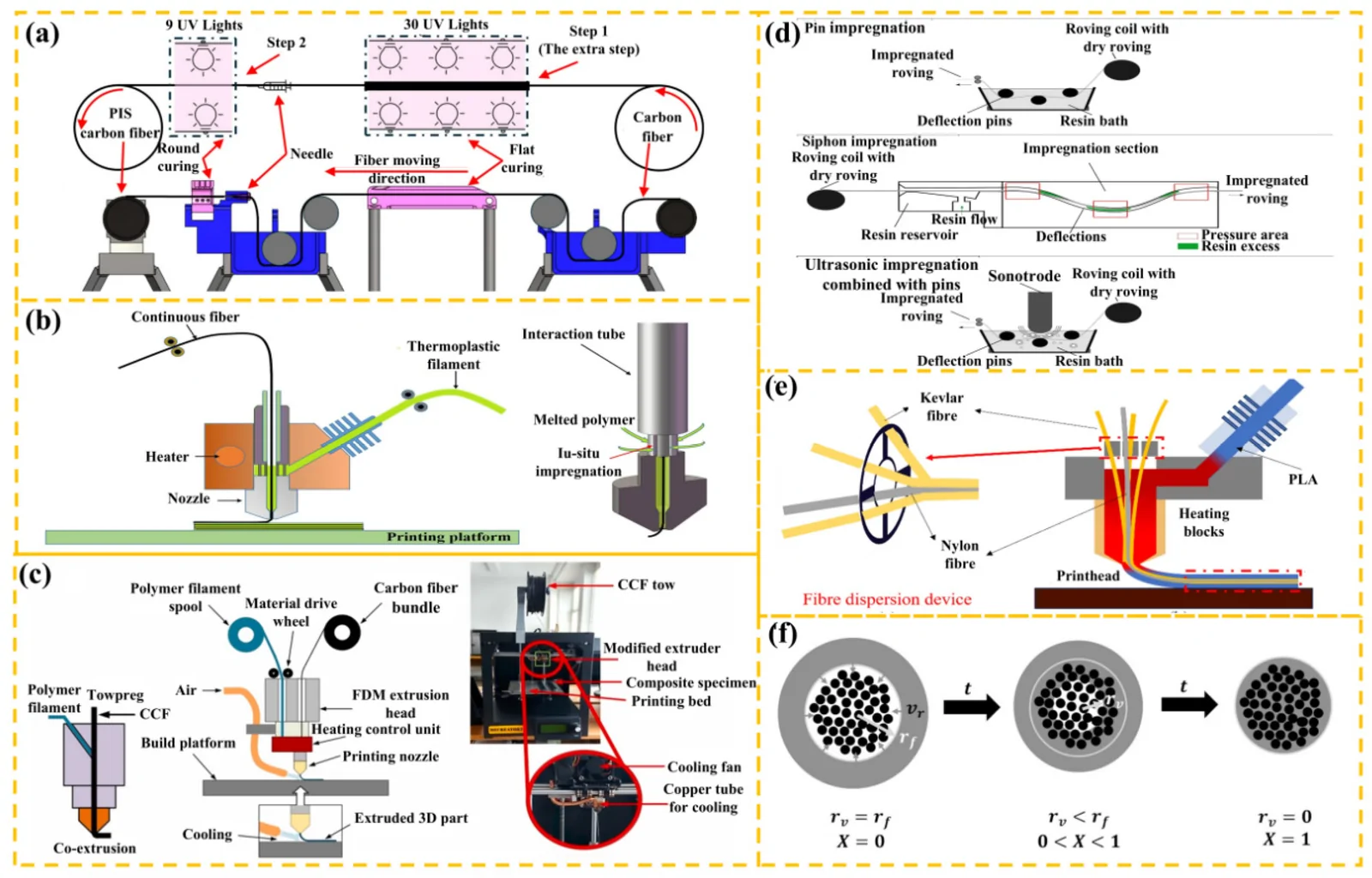
The prepreg preparation methods. (**a**) Preparation of continuous-fiber prepreg via the resin impregnation method [71]. (**b**) Schematic diagram of in situ impregnation of continuous-fiber 3D printing [22]. (**c**) Co-extrusion using the towpreg process [25]. (**d**) Schematic layout of pin impregnation, siphon impregnation and ultrasonic impregnation combined with pins [81]. (**e**) HDI impregnation strategy [86]. (**f**) Geometry of the impregnation process model [87].

Continuous-fiber impregnation is one of the most decisive feedstock-level variables in AM because it governs fiber–matrix adhesion, void evolution, and interlaminar load transfer throughout deposition and consolidation [21,71]. Impregnation should be viewed not as a preliminary material-preparation step, but as a process-critical bridge connecting feedstock design, deposition physics, and structural performance. In current practice, two principal routes dominate: ex situ prepreg/towpreg preparation and in situ impregnation during printing [72]. Prepreg-based routes offer a comparatively well-defined initial impregnation state, more stable resin distribution, more reproducible fiber-volume fractions, and generally better consolidation consistency because resin infiltration is largely established before deposition (Figure 2a) [73]. Related studies regarding continuous carbon-fiber thermoplastic systems, as well as reviews of continuous carbon-fiber AM, show that pre-impregnation remains central when dimensional stability and high mechanical reliability are required [22,74].

By contrast, in situ impregnation introduces dry fiber tows into the deposition head, where impregnation occurs immediately before or during extrusion (Figure 2b) [22,75]. This architecture provides greater adaptability in material combinations, fiber contents, and multifunctional integration while reducing dependence on preprocessed feedstocks [23,76]. Nevertheless, successful in situ impregnation requires precise synchronization among melt viscosity, fiber-bundle permeability, fiber tension, thermal history, applied pressure, and deposition rate, because these variables jointly determine impregnation degree, residual void fraction, and interfacial consolidation [8,77]. Consequently, composite quality in in situ systems is often more sensitive to local variations in wetting behavior and thermal management [24,78].

Co-extrusion with towpreg has emerged as an intermediate solution that combines the stability of prepreg feedstocks with the adaptability of in situ processing (Figure 2c) [25,49]. In this architecture, a pre-impregnated fiber core is co-extruded together with additional molten thermoplastic material, allowing local adjustment of matrix content and improved inter-road bonding during deposition. In co-extrusion architectures, the pre-impregnated core stabilizes the initial fiber–matrix state, whereas the secondary molten matrix can compensate for local resin deficiency and enhance inter-road bonding, thereby improving consolidation consistency, fiber-volume-fraction control, and geometric stability [79]. Consequently, co-extrusion with towpreg is increasingly viewed as a promising route toward high-performance and scalable 3DP-CFRPCs [80].

From a practical manufacturing perspective, the distinction among prepreg-based, in situ, and co-extrusion routes becomes clearer when their performance is considered using common engineering metrics rather than process configuration alone. Across the literature, prepreg-based routes generally provide the most reproducible impregnation state and fiber-volume fraction because resin distribution is established before deposition. This reduces the sensitivity of the printing stage to local wetting fluctuations and typically improves consolidation stability and interfacial consistency. Their principal limitation is that the impregnation burden is shifted upstream to feedstock manufacture, storage, and handling, thereby reducing material flexibility and increasing the complexity of feedstock preparation.

In situ impregnation represents the opposite end of this trade-off. Because dry fiber and molten matrix are combined immediately before or during deposition, the route provides greater freedom in matrix–fiber combinations and local fiber content, but impregnation degree and void fraction become strongly dependent on the instantaneous balance among matrix viscosity, fiber-bundle permeability, fiber tension, temperature, pressure, and deposition speed. Increasing printing speed can further shorten the available wetting and resin-infiltration time, making interfacial quality and interlaminar performance more sensitive to process fluctuations. Thus, although in situ impregnation offers high configurational flexibility, it generally requires tighter process control to achieve the same level of impregnation uniformity and repeatability as pre-impregnated feedstocks.

Co-extrusion with towpreg occupies an intermediate processing regime. The pre-impregnated fiber core provides a relatively stable baseline impregnation state, while the additional molten matrix introduced during co-extrusion enables local adjustment of resin content and promotes inter-road and interlayer bonding. Consequently, co-extrusion can reduce the sensitivity of impregnation quality to short-term process fluctuations relative to fully in situ systems, while retaining greater material and processing flexibility than that of conventional prepreg-based printing. Its practical advantage is therefore not necessarily the maximization of a single metric, but the simultaneous balancing of impregnation degree, void suppression, fiber-volume-fraction control, interfacial bonding, geometric consistency, and process stability.

Direct quantitative ranking of the three routes in terms of interlaminar shear strength (ILSS), print speed, or energy demand remains difficult because reported values are strongly affected by matrix chemistry, fiber architecture, printer configuration, thermal management, specimen geometry, and testing protocol. Nevertheless, the available evidence suggests a useful practical boundary: prepreg-based processing is favored when impregnation reproducibility and structural reliability are prioritized; in situ impregnation is favored when material flexibility and on-demand composition control are more important; and co-extrusion is particularly attractive when a compromise between feedstock consistency, interfacial consolidation, process flexibility, and scalable throughput is required. In this sense, selection among the three routes should be based on a multi-metric process–quality trade-off rather than on impregnation efficiency alone.

Figure 2d illustrates the evolution of impregnation architectures in 3DP-CFRPCs, including pin impregnation, siphon impregnation, and ultrasonic-assisted pin impregnation. Despite their different configurations, these approaches share a common objective: enhancing resin infiltration into dense fiber bundles to improve interfacial bonding and reduce porosity [81]. In conventional pin impregnation, repeated bending and spreading of the fiber tow increase the exposed surface area and facilitate matrix wetting; however, resin penetration into the tow interior remains limited, particularly when processing high-viscosity thermoplastic matrices [82]. Siphon impregnation addresses this limitation by introducing pressure-assisted resin transport, enabling more uniform matrix distribution and improved infiltration efficiency throughout the fiber bundle. Ultrasonic-assisted impregnation has emerged as an effective strategy for further enhancing tow wetting and interfacial consolidation. High-frequency vibration generates localized acoustic streaming and oscillatory pressure fields within the molten polymer, promoting resin mobility, accelerating wetting kinetics, and facilitating the removal of entrapped air [83]. As a result, ultrasonic-assisted systems have demonstrated significantly lower void content and stronger fiber–matrix interfaces compared with the results for conventional impregnation approaches [84,85]. Figure 2e illustrates a hybrid-dispersion impregnation (HDI) approach that improves fiber dispersion and fiber–matrix wetting during in situ printing. By enhancing interfacial contact and reducing microstructural heterogeneity, this strategy promotes more efficient load transfer and improved mechanical performance, highlighting the importance of feedstock architecture design in 3DP-CFRPCs [86].

Impregnation treatment improves the fiber–matrix interface by promoting resin penetration into the fiber bundle, reducing interfacial voids, and increasing the effective contact area between the continuous fiber and the surrounding matrix (Figure 2f) [87]. Under appropriate thermal and pressure conditions, polymer chains can more effectively diffuse into the interphase region, leading to stronger physical entanglement and, in some systems, improved chemical compatibility through enhanced sizing–matrix interaction [50]. As a result, load transfer across the interface becomes more efficient, while crack initiation and interfacial debonding are effectively suppressed. Consequently, impregnation treatment not only increases interfacial adhesion but also improves the overall consolidation quality and structural reliability of 3DP-CFRPCs [88,89].

Within the smart architecture framework, continuous-fiber impregnation should be regarded as a process-governing mechanism rather than as a simple feedstock preparation step. Its function is to regulate resin infiltration, fiber wetting, interfacial bonding, and porosity evolution, thereby establishing direct links between feedstock characteristics and the resulting microstructure and mechanical performance. Impregnation quality is governed by the coupled interactions among matrix rheology, fiber-bundle permeability, temperature history, pressure conditions, and deposition kinematics, all of which collectively determine resin distribution, interface formation, and load-transfer efficiency [90,91]. In particular, emerging process models for co-extrusion and in situ impregnation reveal that manufacturing parameters such as nozzle temperature, applied pressure, polymer viscosity, and printing speed exert a decisive influence on the impregnation degree and residual defect formation—demonstrating that feedstock design and process regulation are inseparable within smart architecture.

### 3.2. Assisted Forming Strategies in Continuous-Fiber 3D Printing

#### 3.2.1. Thermal-Field-Assisted Forming

Thermal-field-assisted forming has become a key strategy for regulating consolidation quality in 3DP-CFRPCs because the transient thermal history at the fiber–matrix and interlayer interfaces governs matrix viscosity, fiber wetting, polymer-chain diffusion, crystallization or solidification, and residual-stress development. Although infrared, microwave, hot-air, and laser heating differ substantially in penetration depth, heating footprint, energy-coupling mechanism, and spatial selectivity, their effects on bonding can be interpreted through a common state variable: the transient interfacial temperature $T_{int}\left(t\right)$. From this perspective, the relevant process variable is not the nominal heater temperature or energy input alone but the peak interface temperature, the duration for which the interface remains within an effective bonding range, the subsequent cooling rate, and the spatial temperature gradient across the deposited road.

**Figure 3:**
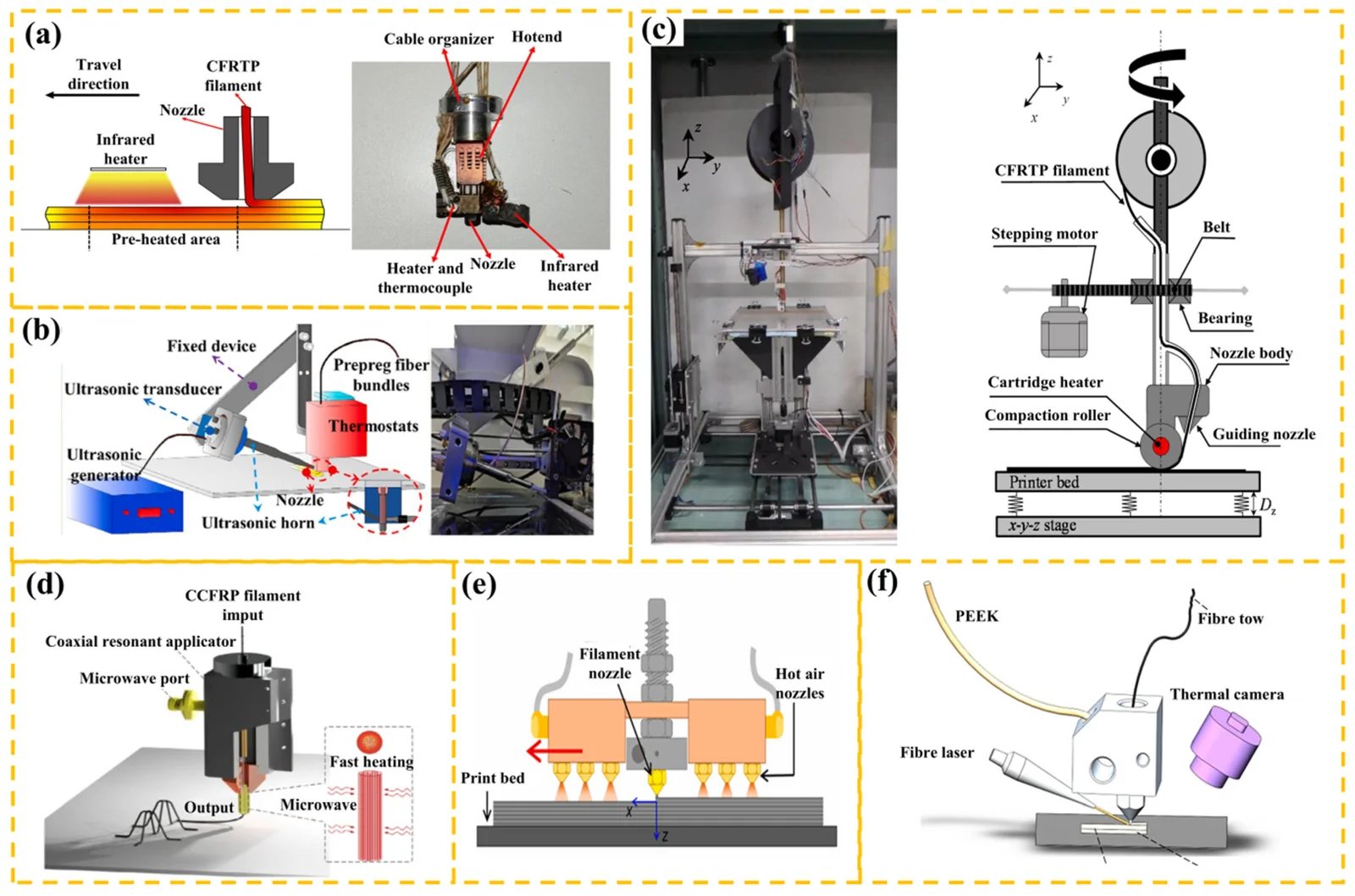
The assisted forming process of 3DP-CFRPCs. (**a**) Infrared [91]. (**b**) Ultrasound [76]. (**c**) Compaction [40]. (**d**) Microwave [93]. (**e**) Hot air [94]. (**f**) Laser-assisted [95].

A generalized thermal-processing condition can therefore be formulated in terms of competing interfacial time scales. During heating, $T_{int}\left(t\right)$ must be sufficiently high to reduce matrix viscosity and promote wetting and molecular mobility. The interface must then remain within this effective bonding range for a time comparable to or longer than the characteristic interdiffusion time required for polymer chains to establish interlayer bonding. For semicrystalline thermoplastics, this diffusion process must occur before cooling and crystallization progressively restrict chain mobility. Conversely, excessive peak temperature or prolonged thermal exposure may lead to uncontrolled resin flow or thermal degradation, whereas steep temperature gradients and rapid non-uniform cooling can generate heterogeneous crystallinity and residual stresses. Thus, an effective thermal-processing window is defined by a balance between viscosity reduction and interfacial diffusion on one side and solidification, residual-stress generation, and thermal degradation on the other. Related studies have shown that infrared heating provides non-contact surface-dominated preheating and can extend the duration for which the deposited interface remains within the effective bonding range, thereby promoting polymer diffusion and reducing the viscosity mismatch between successive roads. However, its limited penetration requires careful control of surface temperature to avoid strong through-thickness thermal gradients (Figure 3a) [91]. 

Among these approaches, microwave heating provides rapid and comparatively volumetric energy delivery (Figure 3d), potentially reducing through-thickness temperature gradients in susceptible material systems. However, its spatial targeting is less precise, and non-uniform electromagnetic coupling can make local $T_{int}\left(t\right)$ more difficult to regulate [92,93]. In contrast, hot-air assistance produces a relatively broad thermal footprint and can prolong the effective bonding time over a larger deposited region, although its lower spatial selectivity may increase the thermal exposure of previously deposited material (Figure 3e) [94].

Laser-assisted heating offers the highest spatial selectivity among the four routes and is especially effective when coupled with compaction pressure and controlled print speed. Laser-assisted heating provides the highest spatial targeting and enables local control of $T_{int}\left(t\right)$, but its concentrated energy input also produces steep spatial and temporal thermal gradients if laser power and printing speed are not appropriately coordinated (Figure 3f). These results indicate that laser heating should be treated not as a standalone heat source, but as part of a coupled thermal–mechanical process window that jointly determines consolidation quality, interfacial integrity, and defect suppression [95]. 

From the smart architecture perspective, the four thermal-assistance routes should therefore not be compared solely according to heater type, nominal power, or maximum temperature. Their common physical descriptor is the path-dependent interfacial thermal history $T_{int}\left(t\right)$, characterized by peak temperature, effective bonding time, cooling rate, and spatial temperature gradient. These quantities jointly regulate matrix viscosity, interfacial diffusion, crystallinity evolution, residual stress, and degradation risk, thereby determining consolidation quality. The practical objective is thus to maintain each material system within a material-specific temperature–time bonding window while minimizing thermal gradients and excessive thermal exposure. Future thermal control should therefore combine in situ interfacial temperature measurement with rheological, diffusion, crystallization, and degradation models to establish transferable processing criteria and enable closed-loop regulation of heating power and deposition speed.

#### 3.2.2. Pressure- and Vibration-Assisted Consolidation

Pressure-, vibration-, and thermomechanical-assisted consolidation strategies address a common limitation of 3DP-CFRPCs: the deposited matrix often exhibits insufficient mobility and insufficient time to establish intimate contact, redistribute resin, eliminate interfacial voids, and develop effective bonding before solidification. Although localized compaction, ultrasonic excitation, post-process hot pressing, and mechanical vibration differ in the manner by which mechanical or thermal energy is introduced, their effects can be interpreted through a common process–state–property relationship. The imposed pressure, contact duration, temperature, and vibration conditions regulate matrix relaxation and resin mobility; these material-state changes determine the degree of interfacial contact and residual porosity, and the resulting interface quality ultimately governs interlaminar load transfer and strength. Therefore, consolidation should be evaluated not only according to the type of external assistance but also according to the balance between the applied consolidation conditions and the relaxation/redistribution behavior of the matrix.

Among various consolidation approaches, compaction-assisted deposition remains the most widely adopted. As illustrated in Figure 3c, rollers or force-controlled compaction devices apply localized pressure immediately after deposition, promoting intimate contact between neighboring roads and layers while suppressing process-induced porosity. The effectiveness of compaction, however, cannot be characterized by pressure alone. A given pressure may produce limited consolidation when the matrix remains highly viscous or when the contact duration is too short for resin redistribution and matrix relaxation. By contrast, the same nominal pressure applied at a temperature associated with higher matrix mobility, or maintained for a longer effective contact time, can promote substantially greater void closure and interfacial contact. This explains why consolidation quality is governed by a coupled pressure–time–temperature condition rather than by maximum force alone. Excessive consolidation severity must also be avoided because high pressure combined with a highly mobile matrix can cause resin starvation, fiber distortion, or loss of geometric fidelity [40].

Ultrasonic-assisted consolidation (Figure 3b) provides an alternative route by introducing high-frequency mechanical vibrations during or immediately after deposition. Unlike conventional compaction, ultrasonic energy enhances molecular mobility at the fiber–matrix interface through localized frictional heating and acoustic softening effects. These mechanisms promote polymer-chain interdiffusion, improve tow wetting, and facilitate resin penetration into densely packed fiber bundles. Continuous carbon-fiber thermoplastic composites have reported substantial reductions in porosity and significant improvements in interlaminar shear strength when ultrasonic vibration is integrated into the deposition process. More importantly, ultrasonic assistance enables localized interface activation without requiring excessive bulk heating, making it particularly attractive for high-performance thermoplastic systems with narrow processing windows [96].

For ultrasonic consolidation, vibration frequency and amplitude should likewise be interpreted through their effects on the local matrix state rather than as independent indicators of consolidation quality. Increasing vibrational input can enhance frictional heating, acoustic softening, polymer mobility, and resin penetration, thereby accelerating void removal and interfacial bonding. However, the useful range is material- and process-dependent: insufficient excitation produces little change in matrix relaxation, whereas excessive vibrational input may generate local overheating, unstable resin flow, or fiber disturbance. Consequently, ultrasonic parameters must be coordinated with interface temperature and exposure time to achieve effective consolidation without introducing secondary defects.

Beyond in situ consolidation, post-process hot pressing illustrates particularly clearly the combined role of pressure, temperature, and consolidation time. Unlike the short contact period available during in situ deposition, hot pressing provides an extended interval in which elevated temperature lowers matrix resistance to flow, while sustained pressure promotes intimate contact and void collapse [97]. The longer available relaxation and redistribution time can therefore substantially improve consolidation, even when defects were generated during the original printing stage. Its limitation, however, is that the improvement is obtained through an additional manufacturing step, and it cannot necessarily correct defects associated with severe fiber misalignment, fiber damage, or poorly designed reinforcement paths [98].

Taken together, the assisted-processing strategies reviewed above occupy different operating regimes rather than form a simple performance hierarchy. Infrared and hot-air heating are attractive when broader and relatively simple thermal conditioning is required, but their lower spatial selectivity limits precise local control. Microwave heating offers rapid volumetric energy delivery, whereas laser assistance provides the highest spatial targeting and is therefore more suitable when the thermal field must follow a localized deposition path; however, this benefit is accompanied by tighter requirements for power, speed, and thermal control. Mechanical consolidation exhibits a similar trade-off. Compaction is comparatively direct and compatible with in situ processing, but excessive pressure can induce fiber distortion or resin starvation, whereas ultrasonic assistance can enhance local matrix mobility and interfacial bonding, without extensive bulk heating, but introduces additional equipment and control complexity. Post-process hot pressing is effective for residual-void reduction and interlayer improvement, but shifts consolidation away from the printing stage and increases the total processing cycle. Consequently, no single assisted-forming strategy is universally superior; the preferred route depends on whether spatial control, throughput, in situ defect suppression, geometric fidelity, or final interlaminar performance is prioritized.

### 3.3. Process-Induced Defects and Structure–Property Relationships

Process-induced defects remain one of the primary barriers preventing 3DP-CFRPCs from achieving the reliability required for structural applications. Importantly, these defects should not be regarded as isolated microstructural imperfections [99,100]. Because impregnation, thermal history, fiber steering, consolidation, and cooling evolve simultaneously during deposition, porosity, fiber waviness, resin redistribution, interfacial degradation, and residual stress frequently develop in a coupled manner. Their mechanical significance, therefore, depends not only on the magnitude of each individual defect, but also on its morphology, spatial location, connectivity, and interaction with neighboring defects [26,101,102].

As summarized in Table 4, porosity is one of the most widely recognized defects, but its mechanical influence cannot be described by void fraction alone. Void size, aspect ratio, orientation, spatial location, and connectivity determine how strongly a defect perturbs the local stress field. Intra-tow pores primarily reduce effective fiber–matrix contact and load-transfer efficiency, whereas elongated inter-road or interlayer voids can generate strong local shear and peel stress concentrations and provide preferential paths for crack coalescence and delamination. Consequently, two specimens with similar total porosity may exhibit substantially different damage tolerance when their void morphologies and spatial distributions differ [103]. Interlayer voids primarily weaken ILSS, fracture toughness, and through-thickness load transfer, thereby promoting delamination under service loading [103,104]. Fiber misalignment and fiber waviness reduce reinforcement efficiency by interrupting the intended load path and increasing anisotropic stress concentration, which is particularly detrimental to tensile modulus, compressive strength, and buckling resistance [105,106,107,108]. In addition, fiber damage or breakage decreases the effective fiber length and compromises load-transfer capability, while residual stresses generated by non-uniform thermal history and cooling shrinkage can induce warpage, distortion, and microcrack formation, further undermining dimensional stability and fatigue performance [109,110,111]. Collectively, these defects demonstrate that the mechanical response of 3DP-CFRPCs is governed not only by fiber-volume fraction, but also by the type, distribution, and severity of process-induced microstructural imperfections [112,113].

More importantly, the defects summarized in Table 4 interact through positive-feedback mechanisms rather than evolving independently. Fiber waviness and local tow misalignment alter the geometry of the consolidation zone and can produce non-uniform contact pressure and resin redistribution [114,115]. These local disturbances may generate matrix-rich and matrix-starved regions; in the latter case, insufficient resin reduces fiber wetting and the effective matrix ligament available for stress redistribution. At the same time, incomplete local impregnation facilitates the retention of intra-tow and interfacial voids. Thus, fiber-path distortion can indirectly amplify both resin starvation and porosity, while porosity itself reduces the effective interfacial contact area and increases the sensitivity of the surrounding matrix to local deformation [116,117]. Residual stress provides an additional coupling mechanism. Non-uniform heating, crystallization, or cooling shrinkage generates spatially heterogeneous residual stresses that are superimposed on the stress concentrations associated with voids, fiber curvature, and matrix-starved interfaces. Elongated or interconnected voids located near a wavy fiber bundle or weak interlayer boundary can therefore experience substantially higher local shear and peel stresses than does an isolated spherical pore in a well-impregnated region. Conversely, local resin starvation and weak interfacial bonding reduce the capacity of the matrix to redistribute these stresses, increasing the probability of microcrack initiation and interfacial separation. The mechanical consequence is therefore controlled by defect co-location and interaction, rather than by any single defect descriptor.

These coupled defect interactions also provide a mechanistic basis for the observed transition in failure mode. When porosity is limited, fiber alignment is maintained, and interfacial bonding is sufficient, damage can remain predominantly matrix-controlled, with distributed matrix yielding or microcracking accommodating local stress concentrations. As voids become larger or more connected, fiber waviness increases, or resin-starved regions weaken the fiber–matrix and interlayer interfaces, the efficiency of stress transfer progressively decreases [118]. Matrix cracks then preferentially propagate toward neighboring voids and weak interfaces, where they can trigger fiber–matrix debonding and interlayer delamination. The dominant failure mechanism therefore shifts from matrix-dominated cracking toward interface-dominated damage. At still higher defect severity, interfacial debonding reduces the effective load-transfer length of the continuous fibers, promoting fiber pull-out, local fiber buckling under compression, or premature fiber fracture under tension [119].

**Table 4:** Typical process-induced defects in 3DP-CFRPCs and their influence on mechanical performance.

Defect Type	Primary Origin	Affected Properties	Typical Influence	References
Porosity	Incomplete impregnation; trapped air; insufficient consolidation	Tensile strength; flexural strength; ILSS; fatigue life	Reduces load-bearing area and acts as stress concentrators, accelerating crack initiation and propagation	[103,104]
Interlayer voids	Insufficient interlayer fusion; inadequate thermal bonding	ILSS; fracture toughness; through-thickness strength	Promotes delamination and weakens interlayer load transfer	[120,121]
Interfacial debonding/poor wetting	Inadequate resin infiltration; insufficient polymer-chain diffusion; poor fiber–matrix compatibility	ILSS; tensile/flexural strength; fatigue resistance	Reduces fiber–matrix load transfer and promotes interfacial crack initiation and delamination	[118,119]
Fiber misalignment	Toolpath deviation; fiber steering; tension fluctuation	Tensile modulus; compressive strength; effective stiffness	Reduces reinforcement efficiency and increases anisotropic stress concentration	[105,106]
Fiber waviness	Deposition instability; geometric constraints	Compressive strength; buckling resistance	Facilitates local instability and premature failure	[107,108,109]
Fiber damage/breakage	Excessive bending, shear, or nozzle-induced abrasion	Tensile strength; fatigue resistance	Decreases effective fiber length and load-transfer capability	[110,111]
Residual stresses	Non-uniform thermal history and cooling shrinkage	Dimensional stability; fatigue performance	Induces warpage, distortion, and microcrack formation	[112,113]

From the smart architecture perspective, process-induced defects should therefore be interpreted as an evolving and spatially coupled defect state rather than as separate independent imperfections. Void morphology and connectivity, fiber waviness, local resin starvation, interfacial integrity, and residual stress jointly regulate the local stress-transfer pathway and determine whether damage remains matrix-dominated or transitions toward interfacial debonding and delamination. This interaction explains why nominally similar porosity levels can produce substantially different mechanical responses when defect morphology and co-location differ. Future process–structure–property models should therefore move beyond single-defect descriptors and incorporate multivariate defect statistics, spatial correlations, and interaction-driven failure mechanisms. In situ imaging, micro-CT, thermal measurements, and full-field strain characterization will be particularly important for resolving these coupled defect states and establishing quantitative links with structural reliability.

## 4. Topology Optimization

Structural design translates material capabilities and manufacturing constraints into optimized load-bearing architectures through the synergistic integration of topology optimization and fiber-path planning. Within the smart architecture framework, geometry and reinforcement trajectories are therefore co-designed to maximize mechanical efficiency without compromising manufacturability.

### 4.1. Fiber Path Optimization

Fiber path optimization has become a central design problem in 3DP-CFRPCs because structural performance depends not only on where material is placed, but also on how the reinforcement is routed through space. In contrast to conventional short-fiber composites, continuous-fiber systems allow reinforcement trajectories to be intentionally aligned with the load path; however, the resulting optimal layout must satisfy not only structural objectives but also explicit manufacturing constraints associated with fiber continuity, minimum steering radius, tow width, local gap/overlap, fiber tension, nozzle accessibility, collision avoidance, and deposition stability [122,123]. These constraints arise because a mechanically optimal fiber orientation field cannot necessarily be realized as a continuous and defect-free deposited tow. Consequently, manufacturability should be embedded directly into the optimization formulation rather than evaluated only after an unconstrained structural solution has been obtained [124].

For a continuous-fiber path, represented by a centerline r(s), several practical manufacturing requirements can be explicitly formulated. First, steering capability imposes a curvature constraint,

(1) $$\kappa \left(s\right)\leq \kappa_{max}=\frac{1}{R_{min}}$$

where $R_{min}$ is the minimum allowable turning radius for a given tow–matrix–nozzle system. Second, tow width introduces local coverage constraints: the spacing between neighboring paths should remain within allowable bounds to prevent excessive gaps or overlaps. Third, the fiber-feed system imposes a tension constraint,

(2) $$T_{min}\leq T_{f}\left(s\right)\leq T_{max}$$

because insufficient tension can promote tow buckling and placement instability, whereas excessive tension may increase fiber damage, path deviation, or interfacial disturbance. Nozzle geometry and multi-axis machine kinematics introduce additional clearance, collision, orientation, and accessibility constraints that determine whether the intended fiber direction can be physically realized. Finally, steering-induced damage should be treated as a coupled process constraint rather than as curvature alone because the onset of waviness, fiber distortion, or breakage depends simultaneously on local curvature, fiber tension, deposition speed, matrix temperature, and consolidation state.

**Figure 4:**
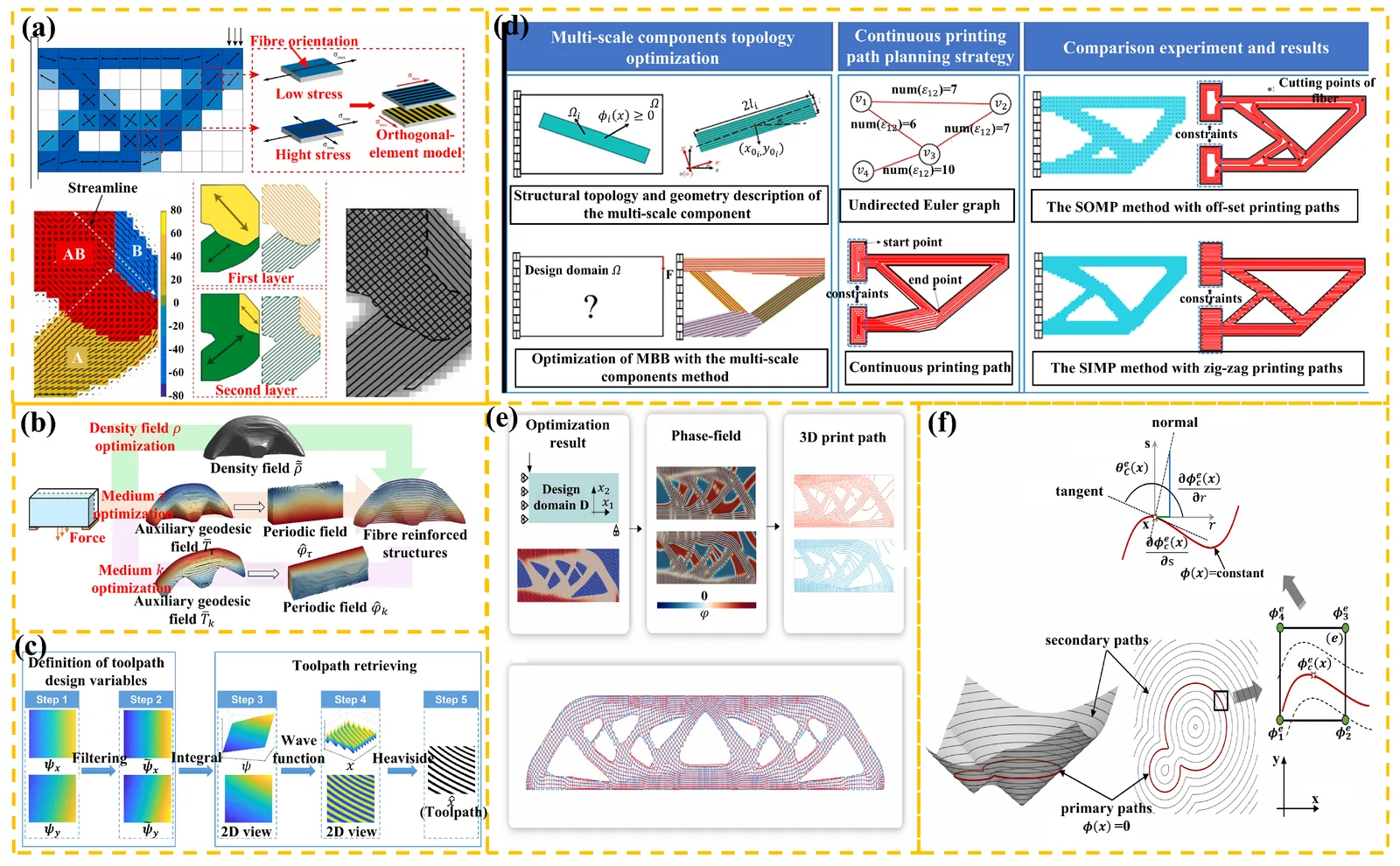
The continuous orientation methods of 3DP-CFRPCs. (**a**) The SOMP printing fiber-path planning strategy [35]. (**b**) DGTO process diagram [136]. (**c**) ISTO [133]. (**d**) Multi-scale components topology optimization method, with the MMC method and continuous-printing-paths planning strategy based on the Euler paths [126]. (**e**) The workflow for TO, high-toughness composite material path design in 3D printing [43]. (**f**) The fiber-path generation based on iso-contours of the level-set function [37].

Figure 4 summarizes representative strategies that capture this transition. In Figure 4a, the solid orthotropic material with penalization (SOMP)-based strategy couples orthotropic material interpolation with density-based optimization so that local fiber orientations can be aligned with principal stress directions, while path compensation is introduced between adjacent layers to preserve continuity [125]. Figure 4d presents a manufacturability-aware multiscale topology optimization framework for continuous carbon-fiber-reinforced composites, in which Moving Morphable Components (MMC)-based structural design is coupled with Euler-path-based printing path planning. This strategy enforces intra-component fiber orientation consistency, improves path continuity, and reduces fabrication defects, leading to marked gains in stiffness and peak load relative to the results for conventional SOMP- and Solid Isotropic Material with Penalization (SIMP)-based path strategies [10,43,126]. Figure 4e illustrates a phase-field-based workflow in which smooth implicit fields are used to extract continuous paths for high-toughness composite printing, naturally enforcing connectivity and curvature continuity while remaining compatible with multi-axis fabrication [127]. Figure 4f illustrates a level-set–based fiber-path generation strategy that converts a topology description into printable fiber trajectories without introducing path discontinuities, sharp turns, or local overlap conflicts. Compared with conventional raster-based infill, level-set-derived paths better preserve continuity and smoothness, which is critical for 3DP-CFRPCs, where fiber breakage, waviness, and excessive curvature associated with an insufficient turning radius can severely degrade load-transfer efficiency [35,128,129,130].

The integrated structure and toolpath optimization (ISTO) (Figure 4c) framework embeds toolpath-width consistency directly into the optimization problem, treating printing feasibility as a first-class design constraint rather than as a post-processing correction [131,132,133]. Likewise, Derivable Geodesics-Coupled Topology Optimization (DGTO) (Figure 4b) introduces geodesic fields to achieve curved-layer generation and continuous-fiber path planning for multi-axis printing, while neural co-optimization frameworks jointly optimize structural topology, manufacturable layers, and path orientations within a single learning-based design pipeline [36,134,135,136]. Together, these studies show that path optimization is no longer a downstream interpretation step after topology design; instead, it is becoming an integral design variable that determines whether a theoretically efficient structure can be realized as a printable continuous-fiber composite [137,138,139].

Of equal importance, manufacturability constraints should be validated against the deposited path rather than assessed only in the digital design space. Experimental verification can be performed by comparing the prescribed fiber trajectory with the as-manufactured centerline using machine vision, optical imaging, or volumetric characterization. Relevant validation metrics include the root-mean-square path deviation, local fiber-orientation error, realized turning radius, tow-width variation, gap/overlap fraction, and the frequency or severity of waviness, buckling, or fiber breakage. Simultaneous monitoring of fiber tension and deposition force can further determine whether predicted process constraints are respected during steering. These path-level measurements should ultimately be correlated with local void content, interfacial quality, stiffness, strength, and failure location so that a numerically optimal trajectory can be distinguished from a genuinely manufacturable and mechanically effective trajectory.

From the smart architecture perspective, fiber-path optimization should therefore be regarded as a constrained translation from structural intent to physical process execution. A mechanically optimal orientation field becomes practically meaningful only when it satisfies explicit limits on curvature, tow coverage, fiber tension, nozzle accessibility, collision avoidance, and steering-induced damage. These constraints should be calibrated for the relevant material–tow–printer system and incorporated directly into topology–path co-optimization. It is also equally importantly that predicted paths be verified against the deposited fiber architecture through quantitative measurements of trajectory error, orientation deviation, realized curvature, tow-width variation, gap/overlap, tension history, and defect formation. Future optimization frameworks should therefore close the loop between structural prediction, machine constraints, deposited architecture, and measured performance, thereby moving continuous-fiber AM from numerically manufacturable designs toward experimentally validated, defect-aware structural synthesis.

### 4.2. Structural Topology Optimization and Multiscale Architected Design

**Figure 5:**
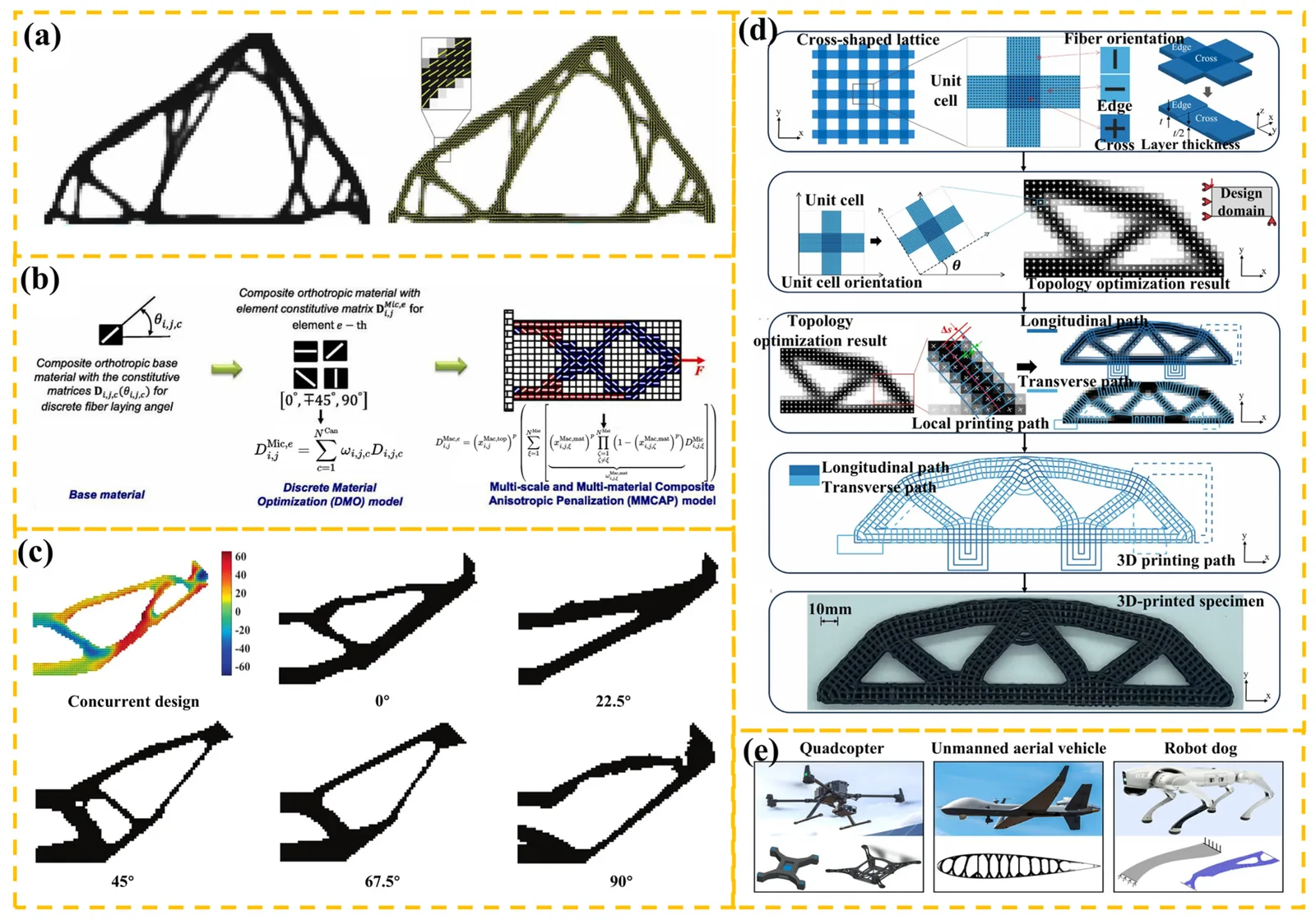
The structural optimization methods of 3DP-CFRPCs. (**a**) Optimization of beading beam with buckling constraint [145]. (**b**) Concurrent multi-material and multi-scale design optimization of fiber-reinforced composite material and structures [150]. (**c**) Optimization of BESO structure with different fiber angles [148]. (**d**) Design of 3D printed TO lattice structures [36]. (**e**) Examples of topology optimization structure applications in lightweight robotic systems [130].

Figure 5a highlights buckling-constrained topology optimization. In slender or load-critical members, stiffness maximization alone is insufficient because instability can dominate failure long before the nominal strength limit is reached [140]. A study on 3D-printed continuous-fiber-reinforced composites explicitly incorporated buckling constraints into topology optimization, showing that structural layout must be designed not only for load-carrying capacity but also for global stability under service loading. This trend is highly relevant for aerospace brackets, thin-walled beams, and robotic load paths, where topology must balance weight reduction against instability suppression [141,142]. 

Figure 5b represents concurrent multi-material and multi-scale design optimization. Instead of treating macro-topology and micro-fiber architecture separately, as does the adaptive normal distribution fiber optimization (ANDFO) scheme, this design combined macro-scale structural topology with micro-scale discrete fiber-laying angle selection, while also introducing nonlinear filtering to preserve manufacturable continuity in the optimized design [143,144,145]. This direction is especially significant for AM because it allows the designer to couple structural layout, local anisotropy, and manufacturing feasibility within a single optimization loop. In the broader literature, similar two-material topology optimization strategies have also been used to couple the design and fabrication of continuous carbon-fiber composite structures, demonstrating that topology optimization can directly support printable, stiffness-enhanced composite architectures rather than abstract numerical layouts [146]. 

Figure 5c further shows how fiber-angle-dependent evolutionary topology optimization has matured into a structural-design tool for anisotropic composites. Bi-directional evolutionary structural optimization (BESO) is effective for maximizing the stiffness of composite laminates while accounting for fiber-angle effects, indicating that discrete reinforcement orientation should be treated as a structural variable rather than as a post-processing detail. Within smart architecture, this is an important conceptual step for 3DP-CFRPCs because it links material anisotropy to structural topology at the design stage [7,147,148]. 

As illustrated in Figure 5d, a lattice design introduces solid-domain topology optimization. A lattice design introduces unit-cell geometry, relative density, and mesoscale connectivity as additional design variables [36]. Multiple investigations of multiscale topology optimization and 3D printing of continuous carbon-fiber-reinforced lattice structures have shown that macro- and mesoscale design can be optimized concurrently to improve stiffness, compression resistance, and structural efficiency [149,150]. Related studies on 2.5D hybrid continuous-fiber lattice structures further demonstrate that hybrid lattice concepts can exploit the tensile efficiency of continuous fibers to enhance compressive performance, underscoring the potential of hierarchical architectures for lightweight load-bearing applications [151]. 

Figure 5e shows that these topology-optimization principles are not limited to academic benchmarks, but are increasingly being transferred to lightweight robotic systems and other application-driven structural components [152]. This reflects a broader shift in the field: topology optimization is no longer used only to remove non-functional mass, but also to generate application-specific architectures in which stiffness, stability, dynamic response, and manufacturability are co-optimized. In robotic and mechatronic systems, this is particularly important because reduced mass directly improves actuation efficiency, positional accuracy, and energy consumption [51,130,153].

An additional challenge is that most topology- and fiber-orientation-optimization frameworks still evaluate a nominal architecture, whereas the mechanically relevant structure is the as-manufactured architecture. In continuous-fiber AM, deviations between these two states are unavoidable because fiber placement, impregnation, consolidation, and thermal evolution are all subject to process variability. As a result, an architecture that is optimal under ideal fiber trajectories, uniform fiber-volume fraction, zero porosity, and perfect interfaces may become highly sensitive to comparatively small manufacturing deviations. Robust design therefore requires manufacturing uncertainty to be incorporated directly into the optimization problem rather than considered only during post-process assessment.

The relevant uncertainties span several structural scales. At the path level, deviations between prescribed and deposited trajectories introduce local fiber-orientation errors and curvature perturbations, which modify the intended anisotropic stiffness field. At the material-distribution level, spatial variations in fiber-volume fraction and local matrix content alter stiffness and load-transfer capacity. At the defect level, variations in void content, morphology, and fiber waviness create local stress concentrations and may shift the onset of damage away from the locations predicted for an idealized structure. Finally, imperfect fiber–matrix and interlayer interfaces introduce uncertainty in shear transfer and delamination resistance. These uncertainties are not independent: path deviation can alter local consolidation, which can in turn affect fiber-volume fraction, porosity, and interfacial quality.

From a robust-design perspective, these manufacturing variations can be represented either as experimentally calibrated statistical distributions or as physically admissible bounds. The optimization objective should then move beyond maximizing nominal stiffness or minimizing nominal compliance toward maintaining acceptable performance over the expected range of manufacturing variability. For example, topology and fiber-path designs may be evaluated simultaneously in terms of mean stiffness, performance variability, buckling reliability, defect sensitivity, and failure probability. Designs that provide slightly lower nominal stiffness but exhibit substantially lower sensitivity to fiber-path error, porosity, or interface degradation may therefore be preferable for practical engineering applications.

Taken together, these developments indicate that structural topology optimization in 3DP-CFRPCs is evolving from deterministic weight- or compliance-oriented design toward multiscale, manufacturability-aware, and uncertainty-aware architecture synthesis. The central challenge is no longer simply to identify a mechanically optimal nominal topology, but to generate an architecture whose performance remains stable after realistic manufacturing deviations are introduced [37,154]. Within the smart architecture framework, topology optimization should therefore incorporate not only stability constraints, anisotropic material allocation, fiber-path continuity, and machine accessibility, but also uncertainties regarding deposited trajectory, local fiber-volume fraction, porosity, fiber waviness, and interfacial properties. Future robust optimization frameworks should combine experimentally calibrated manufacturing variability with structural analysis so that stiffness, buckling resistance, strength, and failure tolerance are optimized for the as-manufactured rather than idealized composite architecture.

## 5. Intelligent Manufacturing

Multifunctional integration elevates continuous-fiber AM beyond structural reinforcement by incorporating sensing, actuation, self-healing, adaptive behavior, and AI-enabled manufacturing intelligence. Within the smart architecture framework, these functionalities converge into a closed-loop paradigm that seamlessly fuses materials, structures, manufacturing processes, and digital intelligence.

### 5.1. Multi-Axis Robotic Manufacturing Platforms

The transition from planar extrusion systems to multi-axis robotic manufacturing platforms has fundamentally expanded the design freedom of 3DP-CFRPCs. Conventional three-axis printing constrains fiber deposition to layerwise trajectories, often forcing discontinuities between optimal load paths and manufacturable tool paths. In contrast, multi-axis systems enable fiber placement along complex three-dimensional geometries, allowing reinforcement architectures to more closely follow principal stress trajectories and thereby improve structural efficiency.

**Figure 6:**
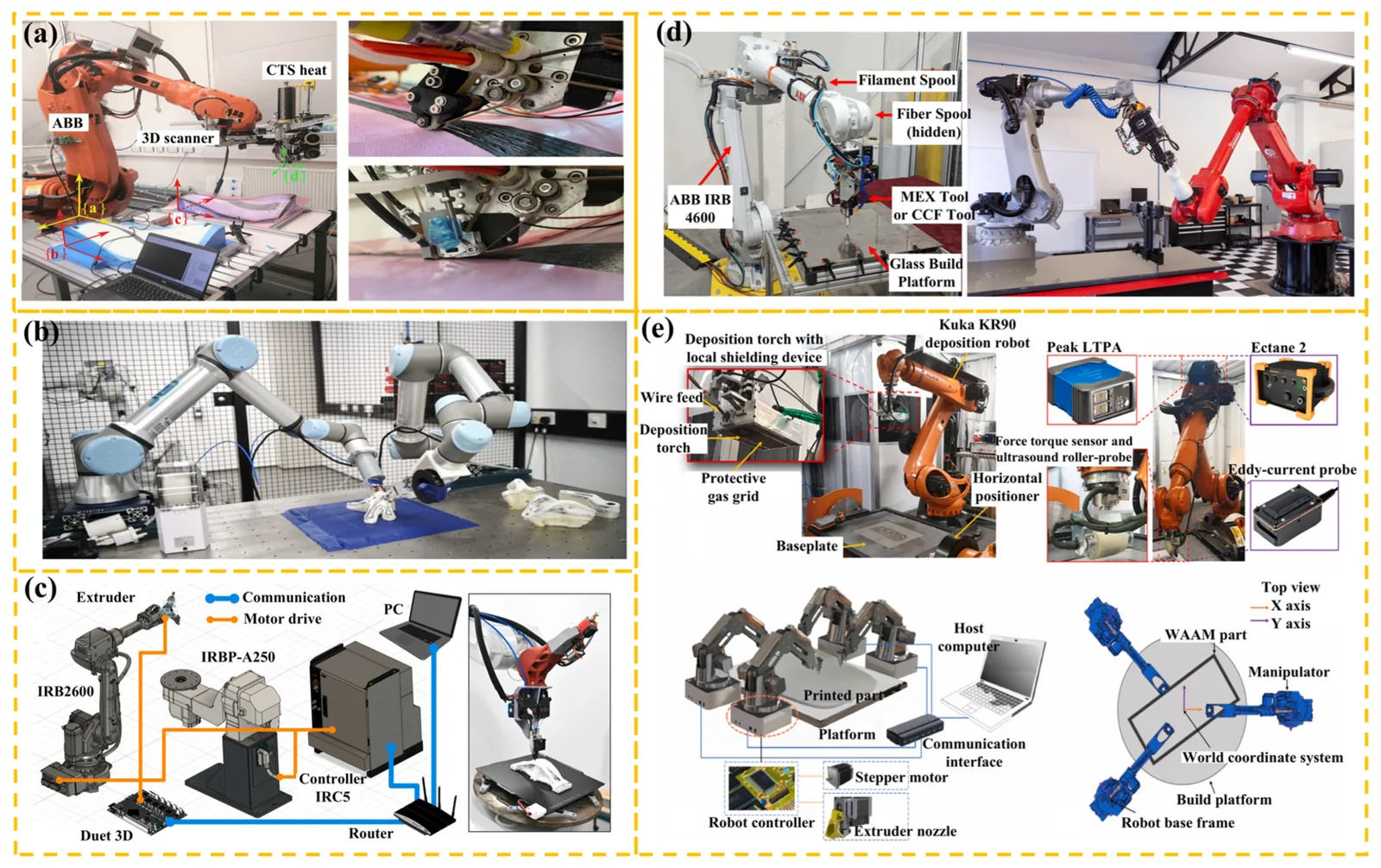
Multi-axis robotic manufacturing of 3DP-CFRPCs. (**a**) Single-arm robotic additive manufacturing (RAAM) systems [155]. (**b**) Dual-arm robotic printing [6]. (**c**) An 8-degree of freedom (DoF) robot-assisted AM system [157]. (**d**) Multi-axis material-extrusion (MEX) robotic deposition platform [88]. (**e**) Multi-arm C-RAAM system [12].

Early robotic implementations employed single robotic (Figure 6a) manipulators equipped with continuous-fiber deposition heads, providing greater workspace flexibility and geometric accessibility than those obtained from conventional gantry-based systems. However, their capability remained limited by restricted process integration and the difficulty of simultaneously controlling fiber placement, material delivery, and support generation [125,155]. To overcome these limitations, dual-arm architectures (Figure 6b) were subsequently developed, enabling the decoupling of matrix deposition and fiber placement while facilitating synchronized toolpath execution. Such systems have demonstrated enhanced control of fiber orientation, reduced geometric constraints, and improved adaptability for complex freeform structures [156].

Figure 6c–e show high-degree-of-freedom manufacturing platforms that integrate robotic manipulators, rotary positioning stages, and multiple deposition heads. These architectures provide additional flexibility for continuous-fiber steering, variable-angle deposition, and support-free fabrication of non-planar geometries [12,128,157]. More importantly, they enable localized regulation of fiber orientation and reinforcement distribution, creating new opportunities for topology-informed structural design and functionally graded composite architectures [88]. At the system level, multi-robot collaborative manufacturing represents the cutting-edge paradigm for continuous-fiber AM. In this configuration, robots are distributed across deposition, fiber placement, in situ inspection, and post-processing, collectively forming a manufacturing ecosystem that simultaneously tackles productivity, geometric complexity, and reliability. These multi-axis platforms serve as the physical execution layer of the smart architecture, embodying the principle that design and manufacturing must be co-optimized. Complemented by force sensing, machine vision, and real-time monitoring, the system further achieves closed-loop adaptability, enabling real-time responses to process disturbances and thereby safeguarding structural integrity [5,158].

For robotic manufacturing platforms, increasing the number of controlled axes expands geometric accessibility and enables fiber trajectories to follow three-dimensional load paths more closely. However, this benefit is accompanied by increasing calibration burden, collision-avoidance complexity, synchronization requirements, and sensitivity to kinematic error. Three-axis systems remain attractive for geometrically simpler and high-throughput deposition, whereas multi-axis or multi-robot platforms become advantageous when non-planar reinforcement, spatially varying fiber orientation, or support-free deposition is essential. The practical transition to higher degrees of freedom is therefore governed by whether the structural benefit of improved fiber placement outweighs the additional planning and control complexity.

### 5.2. Self-Sensing, Structural Health Monitoring and Autonomous Damage Management

Recent advances in 3DP-CFRPCs have driven a paradigm shift from passive load-bearing structures toward intelligent composite architectures capable of simultaneously perceiving, diagnosing, and mitigating damage. Unlike conventional structural composites that require externally attached sensors and offline inspection [159], 3DP-CFRPCs increasingly exploit the intrinsic conductivity of continuous carbon fibers to establish integrated sensing networks embedded directly within the load-bearing architecture. Consequently, the reinforcement phase no longer functions solely as a mechanical constituent but also serves as an information carrier that enables the real-time monitoring of structural integrity throughout both manufacturing and service life [160,161]. 

**Figure 7:**
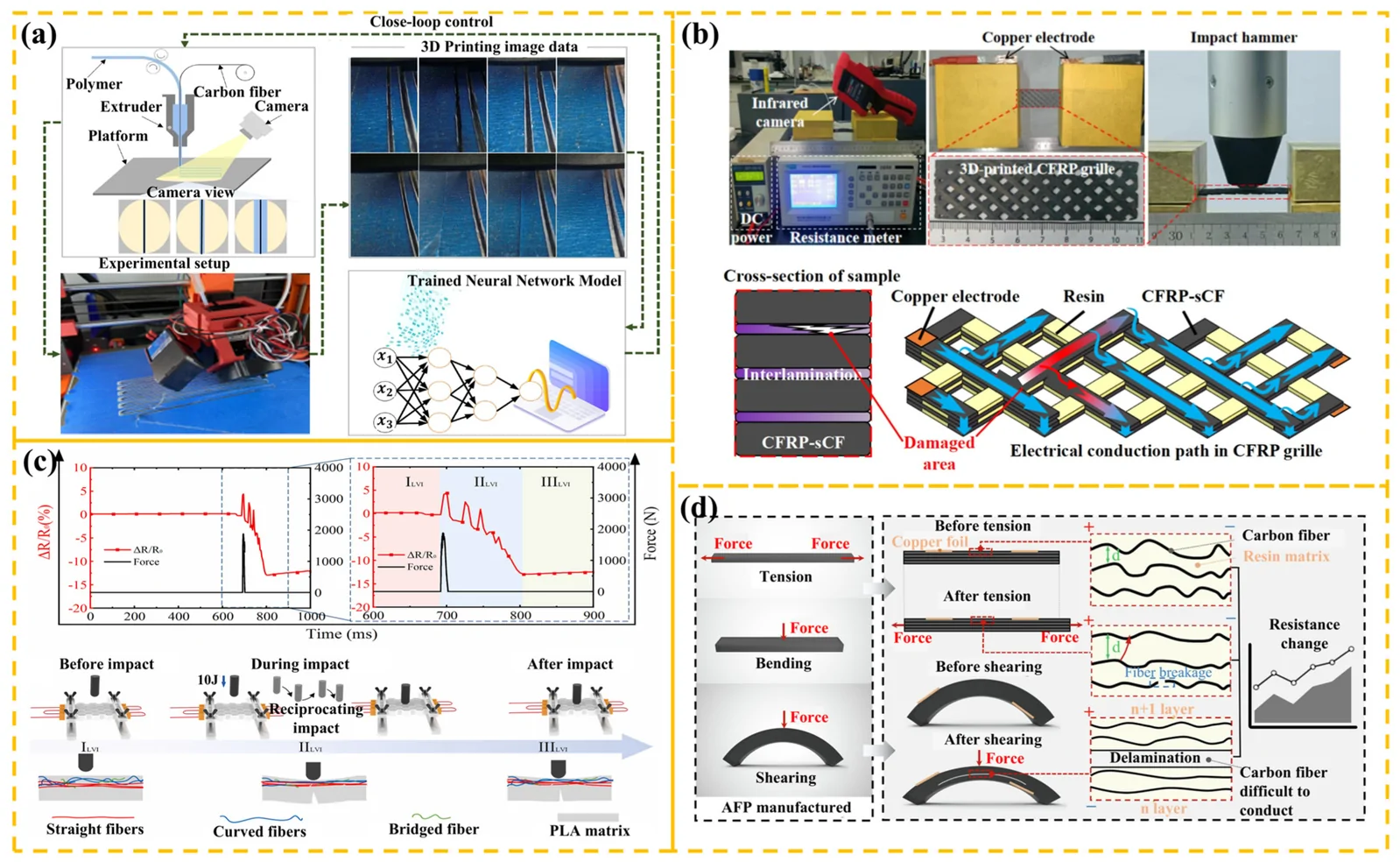
Self-sensing and structural health monitoring of 3DP-CFRPCs. (**a**) Global strategy for online monitoring and feedback control of the CFRPC 3D printing process [174]. (**b**) Self-sensing and self-repairing system for the CFRPC grille [170]. (**c**) Self-sensing behavior of CFRPC honeycomb: electrical resistance changes and diagram of matrix and fiber changes during the impact process [173]. (**d**) The main sensing principle based on automatic fiber placement (AFP) for structural health monitoring [171].

Figure 7 illustrates the evolution of self-sensing architectures in continuous-fiber-reinforced composites. As shown in Figure 7a, a growing number of studies of online monitoring and feedback-controlled printing establish a closed-loop manufacturing framework in which process signals, thermal histories, and electrical responses are continuously collected and used to regulate deposition quality in real time. This capability transforms quality assurance from post-process inspection into in situ process control, providing a critical foundation for intelligent manufacturing of continuous-fiber-reinforced composites. At the structural level, the conductive fiber network can function as a distributed sensing medium [162,163]. Figure 7b demonstrates the characteristic self-sensing response of a CFRPC honeycomb during impact loading. Variations in electrical resistance correlate closely with matrix cracking, fiber/matrix debonding, and progressive damage accumulation, revealing that electrical signals can effectively encode the evolution of internal structural degradation. Unlike conventional point sensors, this distributed sensing mechanism enables continuous tracking of damage propagation while preserving the mechanical integrity of the structure [164].

Beyond damage detection, the existing literature has increasingly integrated sensing and repair functionalities within a unified architecture. As illustrated in Figure 7c, conductive CFRP lattice structures can simultaneously perform damage identification and healing-state evaluation. In such systems, electrical resistance serves not only as a damage indicator but also as a quantitative metric for assessing healing effectiveness. This transition from passive monitoring to active damage management represents a significant step toward autonomous composite systems capable of self-awareness and adaptive response. Under smart architecture, this integration exemplifies the framework’s core proposition: sensing, healing, and load-bearing functions are not add-ons but are co-designed as intrinsic system capabilities. The integration of sensing functionality is also extending into manufacturing platforms [165,166,167]. Figure 7d shows structural-health-monitoring strategies based on automated fiber placement (AFP), where embedded sensing pathways provide real-time information regarding fiber alignment, consolidation quality, and interfacial integrity during fabrication. Such process-integrated monitoring enables early detection of manufacturing-induced anomalies and establishes a direct connection between process quality and long-term structural reliability [13,168,169].

**Figure 8:**
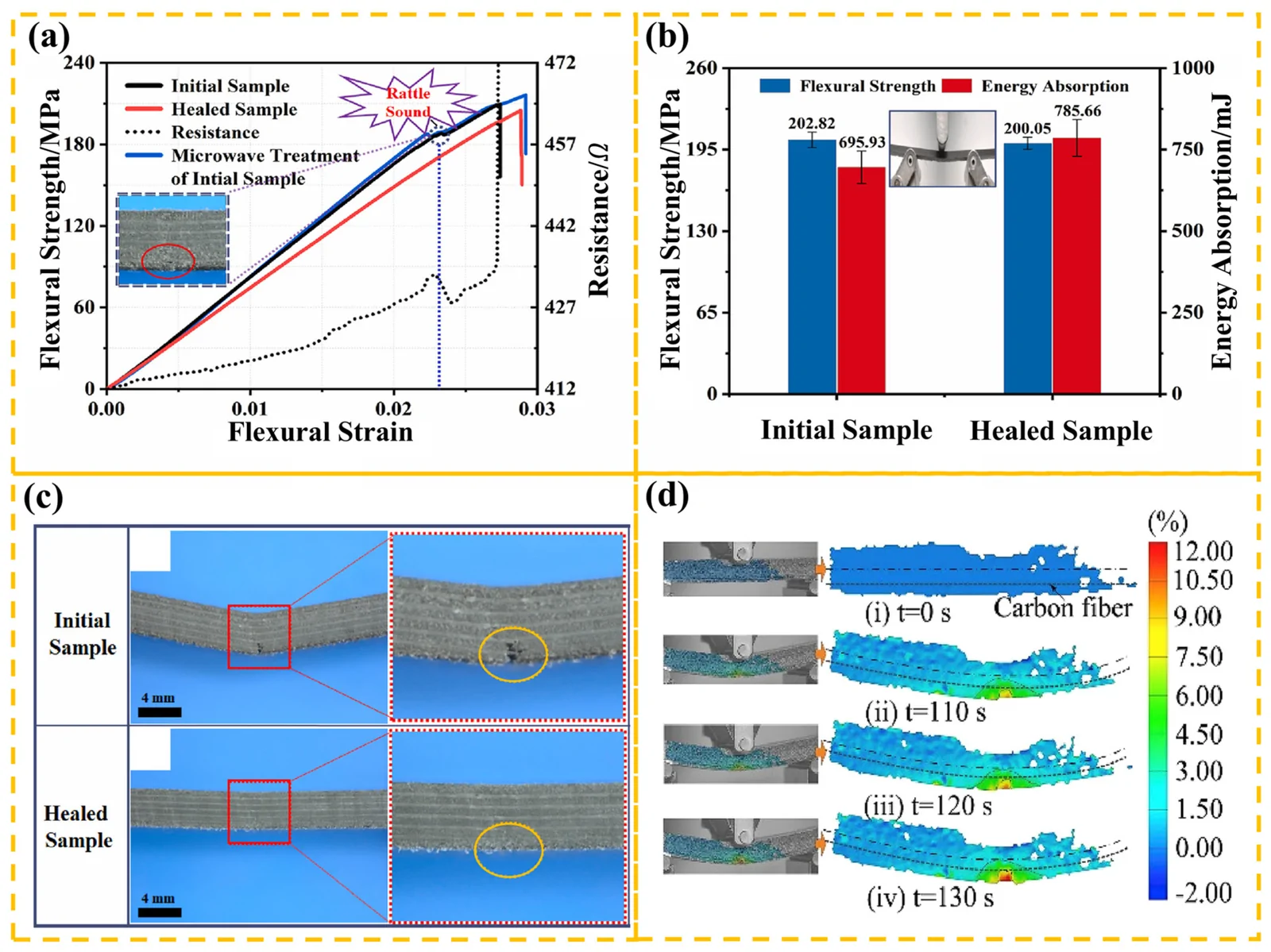
Self-healing characteristics of 3DP-CFRPCs. (**a**) Stress-strain curves of the samples; fiber resistance [160]. (**b**) Strength and energy absorption before and after healing [160]. (**c**) Morphology of the sample before and after healing [160]. (**d**) DIC results of full-field flexural strain distribution for self-healed specimen with continuous carbon-fiber tow [172].

The ultimate objective of intelligent composites, however, extends beyond damage diagnosis toward autonomous performance recovery. Figure 8 highlights progress in self-healing continuous-fiber-reinforced composites and demonstrates how self-sensing architectures can be coupled with damage-repair mechanisms to form closed-loop damage-management systems. As shown in Figure 8a, the stress–strain response of the healed specimen closely approaches that of the pristine material, while the evolution of fiber resistance provides direct evidence of damage accumulation and subsequent structural restoration. Prior to healing, electrical resistance increases significantly due to crack formation and interfacial debonding. Following microwave-assisted healing, resistance fluctuations are substantially reduced, indicating the re-establishment of conductive pathways and improved interfacial continuity [170]. The quantitative results presented in Figure 8b further demonstrate the remarkable effectiveness of the healing process. Flexural strength recovery exceeding 95% and energy-absorption recovery surpassing 110% indicate that healing not only restores the original load-bearing capability but can also enhance post-damage toughness through crack closure, matrix remobilization, and improved stress redistribution. Such recovery levels significantly exceed those typically observed in conventional thermoset-based composite systems [160,171]. These high single-cycle recovery values demonstrate the potential of the healing mechanism, but they should not be interpreted as evidence of long-term healing durability without repeated damage–healing validation.

Microstructural observations provide additional insight into the underlying mechanisms. As shown in Figure 8c, matrix cracks and fiber–matrix interfacial separations present in the damaged state become substantially reduced after healing. The reformation for the fiber-bridging regions suggests that the healing process operates simultaneously at both the matrix scale and the fiber–matrix interface, thereby restoring efficient load transfer across multiple structural hierarchies [172]. Furthermore, full-field strain analysis using digital image correlation (Figure 8d) reveals a more uniform strain distribution after healing, indicating effective recovery of load-transfer continuity and suppression of local strain concentrations. Although residual heterogeneity remains in the previously damaged regions, the overall strain field approaches that of the undamaged structure, confirming the functional restoration of structural integrity [173,174].

Despite these promising demonstrations, reliable structural health monitoring requires more than a measurable electrical response to damage. A primary concern is the stability of the sensing baseline and calibration over time. Electrical resistance in continuous carbon-fiber networks can respond not only to irreversible damage but also to reversible strain, temperature variation, interfacial evolution, and accumulated loading history. Consequently, calibration drift can progressively alter the relationship between resistance change and structural damage, while temperature-dependent electrical and matrix behavior may introduce apparent signal changes that are unrelated to damage. Reliable implementation therefore requires temperature compensation, baseline tracking, and periodic or adaptive recalibration so that environmental and service-induced signal variations can be separated from genuine structural degradation [175]. 

A second requirement is cyclic repeatability and damage-signal discrimination. For a sensing architecture to support long-term monitoring, its sensitivity should remain reproducible over repeated loading–unloading cycles, with limited hysteresis, baseline drift, and cycle-dependent degradation. More importantly, reversible electromechanical responses associated with elastic deformation should be distinguished from irreversible signal changes produced by matrix cracking, fiber–matrix debonding, fiber fracture, or delamination. This sensing–damage decoupling is critical because an electrical signal alone does not uniquely identify the underlying damage mechanism. Combining resistance measurements with strain, temperature, imaging, or other independent sensing modalities can therefore provide a more robust basis for damage-state identification and reduce the risk of interpreting normal thermomechanical variations as structural damage [176]. 

The same reliability requirement applies to self-healing. The high strength and energy-absorption recovery reported after a single healing event demonstrates functional recoverability, but it does not by itself establish service durability. Repeated damage–healing cycles may progressively alter matrix mobility, interfacial integrity, conductive pathways, and the amount of recoverable damage. Healing performance should therefore be characterized in terms of the retention of mechanical strength and stiffness, recovery of the sensing baseline, accumulated residual damage, and healing efficiency over multiple cycles rather than by single-cycle recovery alone [167,177]. 

Finally, reliable structural health monitoring (SHM) requires a clearly defined minimum detectable damage (MDD). The relevant threshold should be linked to the smallest physically meaningful damage state that produces a signal distinguishable from measurement noise, temperature-induced variation, cyclic hysteresis, and calibration drift. Depending on the application, MDD may be expressed in terms of crack length, delamination area, impact severity, fiber-breakage extent, or another physically measurable damage descriptor. Calibration against independent techniques such as imaging, full-field strain measurement, or volumetric inspection is therefore essential for converting a resistance change from a qualitative damage indicator into a quantitatively interpretable structural-health metric [178,179]. 

Taken together, these developments indicate that integrated sensing and self-healing can provide the functional basis for autonomous damage management, but reliable SHM requires a transition from demonstration-oriented to qualification-oriented metrics. Sensing architectures should therefore be evaluated in terms of calibration stability, temperature sensitivity, cyclic repeatability, damage selectivity, and minimum detectable damage, while self-healing architectures should additionally be assessed for multi-cycle recovery and residual-damage accumulation. Within smart architecture, these quantities represent system-level reliability variables linking material functionality, sensing, structural damage, repair, and control. Future closed-loop systems should integrate signal compensation, multimodal damage identification, healing activation, and post-healing verification so that autonomous intervention is triggered by a validated structural state rather than by a single uncorrected sensing signal.

### 5.3. Shape Control and Programmable Morphing in 4D-Printed Continuous-Fiber-Reinforced Composites

The integration of continuous fibers into 4D printing has expanded AM from static part fabrication toward time-dependent, stimulus-responsive architectures capable of programmable shape change. In these systems, the continuous-fiber network serves a dual role: it reinforces the structure mechanically while also tailoring the local stiffness field, deformation anisotropy, and actuation pathway [180,181]. As a result, shape morphing is no longer governed solely by the matrix response, but emerges from the coupled interaction among fiber routing, interfacial continuity, and stimulus-activated matrix softening [182,183]. Multiple investigations on 4D-printed continuous-fiber-reinforced composites have shown that such architectures can realize deterministic, repeatable, and directionally controlled transformations under thermal, electrothermal, or magnetoelectro stimuli, establishing programmable morphing as a distinct class of structural–functional integration in 3DP-CFRPCs [184]. 

**Figure 9:**
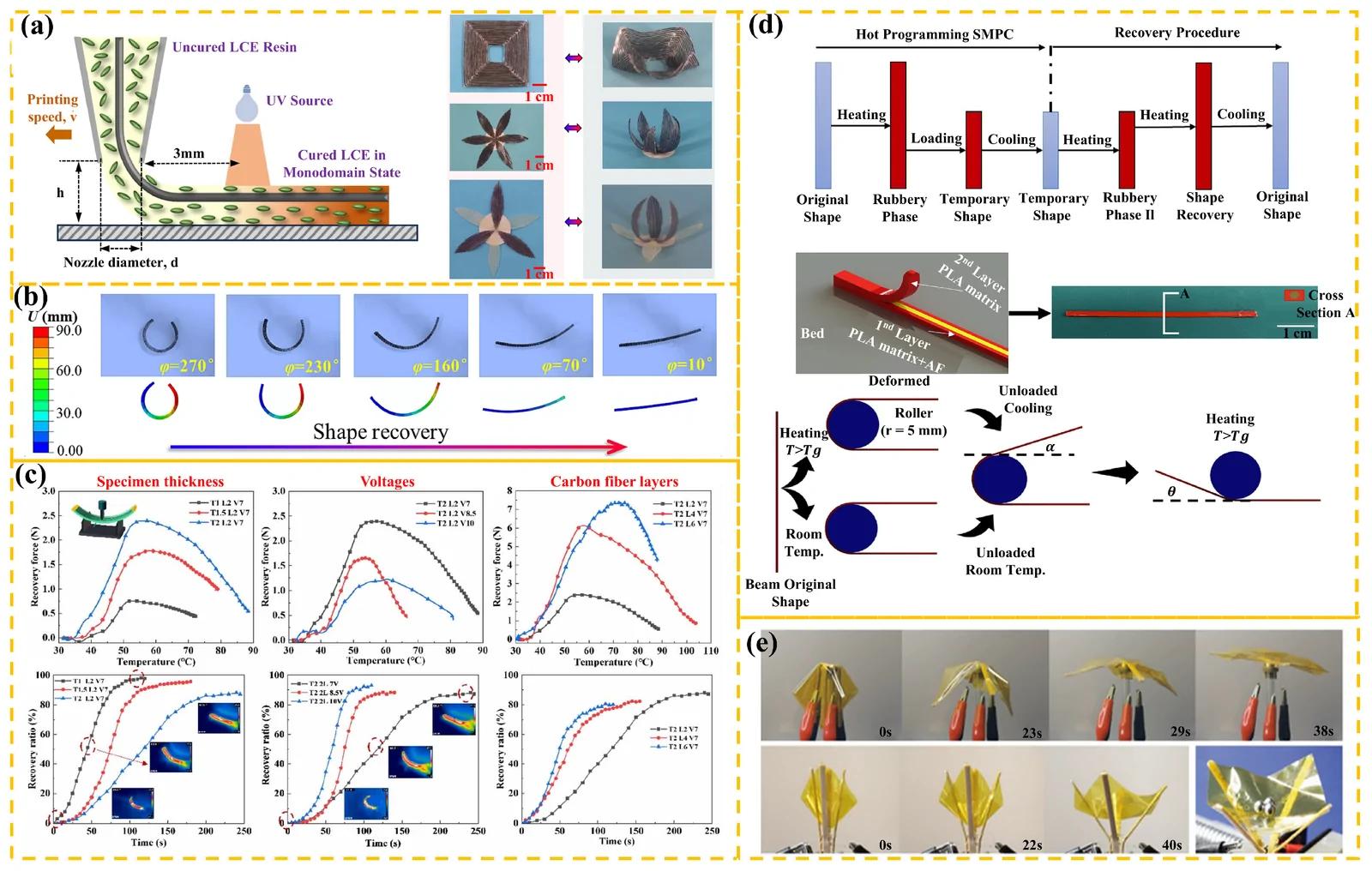
4D shape morphing of 3DP-CFRPCs. (**a**) 4D printing of LCE composites with continuous fiber and their reversible shape-changes upon heating [186]. (**b**) Experiments and simulations of the shape recovery process for the rectangular specimen [197]. (**c**) Shape memory, temperature, shape recovery rate, and time curves [199]. (**d**) 4D printing of bio-shape memory polymer composites, shape programming, and recovery [196]. (**e**) Example of 4D-printed biomimetic gripper with expandable joints based on shape-memory PEEK [198,203].

Figure 9a highlights one of the most important emerging directions: liquid-crystal elastomer (LCE) composites reinforced with continuous fibers. In contrast to conventional thermoplastic or thermoset systems, LCEs provide an intrinsically responsive matrix that can reversibly convert thermal input into macroscopic deformation [185,186]. When combined with continuous-fiber reinforcement, the actuation response becomes more spatially programmable because the fiber architecture constrains local strain evolution and defines the preferred bending or twisting loci [187,188]. This allows a single printed part to undergo large, reversible, and geometry-preserving shape changes upon heating, while maintaining sufficient structural integrity for practical deployment [14,189,190]. The resulting behavior demonstrates that fiber-reinforced LCEs are not simply soft actuators, but architected morphing systems in which actuation direction, amplitude, and reversibility are all encoded by the printed architecture [191]. 

A second important trend is the development of shape-memory polymer composites (SMPCs). With continuous fibers, the shape-memory effect is governed by thermally triggered recovery of a programmed temporary configuration, but the recovery kinetics, force generation, and recovery completeness are strongly modulated by fiber content, specimen thickness, and thermal activation conditions [192,193]. Figure 9b further shows that experiments and simulations can capture the recovery trajectory of a rectangular specimen with good agreement, highlighting the feasibility of model-guided design for predicting recovery behavior [194,195,196,197]. The curves in Figure 9c indicate that shape recovery rate and recovery time are highly sensitive to temperature and structural design, confirming that actuation performance must be optimized as a coupled thermomechanical problem rather than as a material-only property [198,199]. In Figure 9d, bio-inspired SMPCs demonstrate that shape programming and recovery can be controlled through the printed architecture, enabling recoverable morphologies that are difficult to realize by conventional molding. Collectively, these results underscore that continuous fibers act not only as reinforcements, but also as structural regulators that shape the stored elastic energy, recovery pathway, and functional stability of the printed composite [200]. 

Figure 9e illustrates a biomimetic gripper with expandable joints based on shape-memory PEEK, showing how 4D printing can convert continuous-fiber-reinforced composites into load-bearing soft robotic components with controllable opening and closing behavior [196,201,202,203]. Such demonstrations are significant because they bridge the gap between laboratory-scale morphing specimens and application-oriented deployable systems [204,205]. From a smart architecture perspective, the key scientific issue is not merely whether a composite can recover its shape, but how the fiber architecture, matrix chemistry, and triggering mode should be co-designed so that morphing remains repeatable, efficient, and mechanically reliable under service conditions [206,207]. Therefore, 4D printing of continuous-fiber-reinforced composites should be viewed as an architected multi-physics platform in which structural reinforcement and temporal actuation are jointly programmed during fabrication, opening a pathway toward adaptive grippers, deployable structures, soft robotic elements, and reconfigurable load-bearing systems [38].

### 5.4. AI-Enabled Sensing-to-Control Hierarchy for Intelligent Manufacturing

Artificial intelligence (AI) is progressively expanding the role of process data in 3DP-CFRPC manufacturing, with applications ranging from sensing-assisted monitoring and offline defect recognition to predictive modeling, digital-twin state estimation, process optimization, and, more recently, real-time closed-loop control. These capabilities represent distinct levels of manufacturing intelligence and should not be interpreted as technologically equivalent [208]. In this review, AI-enabled manufacturing is therefore organized as a six-stage sensing-to-control hierarchy: (i) sensing and data acquisition, (ii) defect detection and classification, (iii) surrogate/process–property modeling, (iv) digital-twin state estimation, (v) process optimization and decision support, and (vi) real-time closed-loop and autonomous control. Progression through this hierarchy requires increasingly tight integration among physical sensing, model inference, decision making, and actuator-level process correction [64,174].

**Figure 10:**
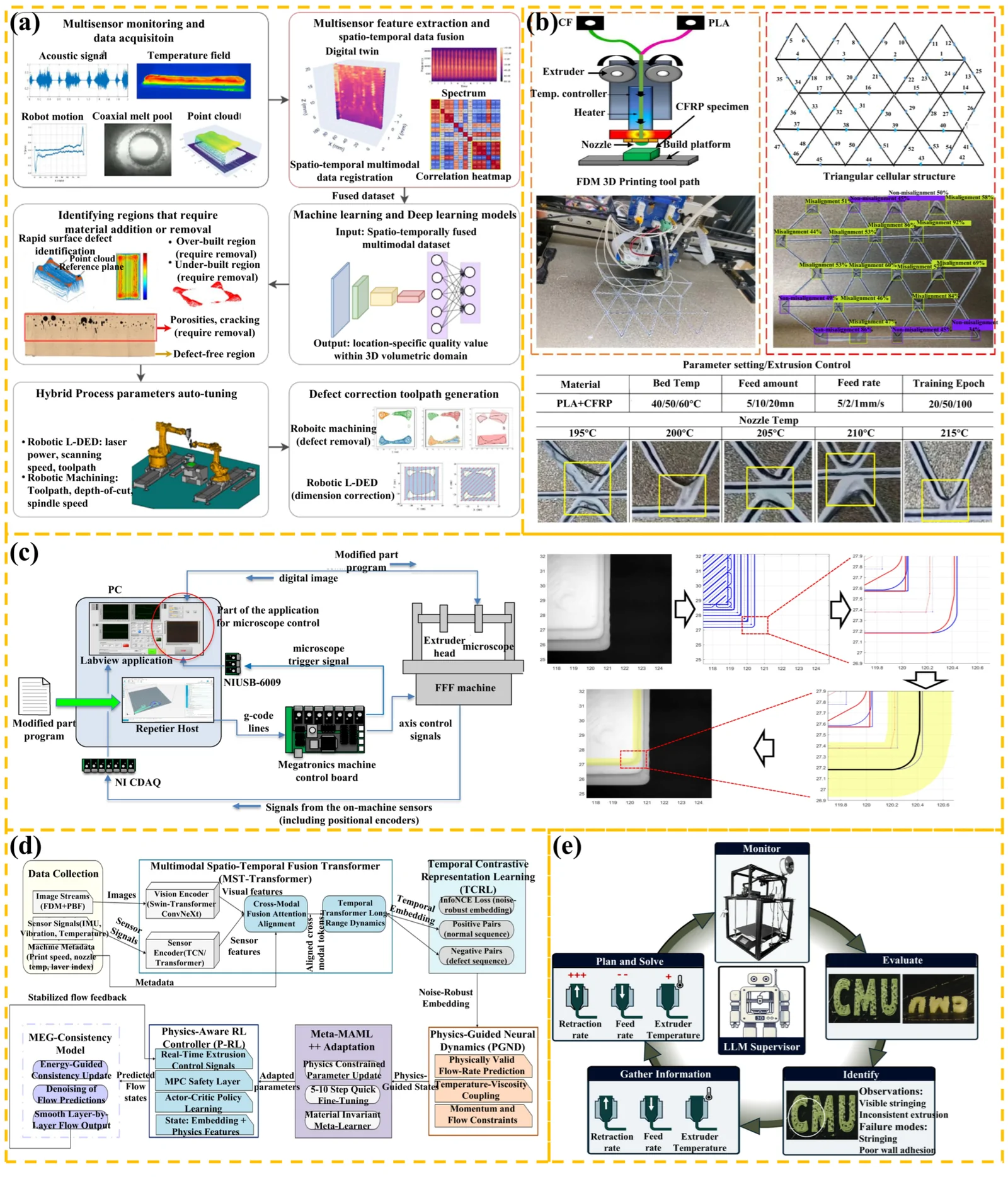
AI-enabled monitoring and autonomous control in 3DP-CFRPCs. (**a**) Multisensory digital twin in robotic LDED AM for in situ quality-monitoring based on the data [33,212]. (**b**) YOLOv8 misalignment defect-detection accuracy [210]. (**c**) Contour-edge detection in AM process [209]. (**d**) MPACT-3D framework for autonomous 3D printing material-flow control [215]. (**e**) LLM-3D print [218].

(i) Sensing and data acquisition. The first level of intelligent manufacturing is process observability. Thermal imaging, machine vision, force sensing, vibration monitoring, electrical measurements, and machine-state acquisition provide complementary information about temperature evolution, tow placement, material flow, consolidation, and geometric fidelity. These sensing modalities are particularly important for 3DP-CFRPCs because the printing process simultaneously involves thermal transport, matrix rheology, fiber tension, impregnation, and path-dependent deposition. However, sensing alone does not constitute intelligent or closed-loop control; rather, it provides the physical data required by subsequent perception, prediction, and decision-making layers. Multimodal sensing is therefore valuable primarily because it increases the observability of coupled process states that cannot be inferred reliably from a single signal.

(ii) Defect detection and classification. The second level converts sensor data into manufacturing information. Machine-vision and deep-learning methods can identify fiber-placement errors, geometric deviations, extrusion instability, and other visible process anomalies. YOLO-based misalignment detection has been investigated directly for continuous carbon-fiber-reinforced polymer printing, demonstrating the feasibility of automated recognition of fiber-placement defects (Figure 10b) [209,210]. By contrast, contour-edge monitoring and digital-twin-based geometry tracking developed for conventional fused filament fabrication (FFF) provide transferable methodologies for dimensional monitoring but do not, by themselves, validate continuous-fiber-specific defect classes such as tow waviness, incomplete impregnation, or interfacial discontinuity (Figure 10c) [211]. Generic vision architectures therefore require retraining and validation against continuous-fiber-specific defect states before their reported accuracy can be extrapolated to 3DP-CFRPCs. More importantly, real-time defect detection should be distinguished from closed-loop manufacturing: a model remains a monitoring or perception system unless its output is translated into a corrective action and physically executed during printing.

(iii) Surrogate and process–property modeling. A third level of intelligence involves predictive models that relate manufacturing variables and sensor features to hidden material states or performance indicators. Machine-learning regressors, physics-informed surrogate models, and hybrid data-driven approaches can be used to establish rapid mappings among temperature, extrusion conditions, pressure, printing speed, geometric features, defect probability, and mechanical performance. Such models are valuable for sensitivity analysis and parameter selection because they reduce the computational cost of repeated high-fidelity simulations or experiments. Nevertheless, most surrogate models remain offline or supervisory unless they are continuously updated using in-process data and coupled directly to process correction. Accordingly, offline regression or machine-learning-based process optimization should be classified as exhibiting predictive or decision-support capabilities rather than as autonomous manufacturing systems.

(iv) Digital-twin state estimation. Digital twins represent a higher level of integration because they require dynamic synchronization between the physical manufacturing process and a virtual representation. The multisensor digital-twin example shown in Figure 10a was developed in the broader additive-manufacturing field rather than specifically for continuous-fiber composite printing [212]. Its relevance to 3DP-CFRPCs therefore lies in its transferable architecture of synchronized multimodal sensing, virtual-state estimation, and feedback-oriented decision making rather than as a direct validation of fiber impregnation, tow transport, or interfacial consolidation. A fiber-specific digital twin would need to estimate additional states such as interfacial temperature, tow tension, fiber-placement deviation, impregnation degree, void evolution, and consolidation quality. This distinction is important because a digital twin should not be equated with a static simulation or an offline machine-learning model; equally, state estimation alone does not constitute autonomous control unless the estimated state is subsequently used to generate and execute corrective process actions. Continuous-fiber-specific studies of online monitoring, multisensor fusion, and intelligent process regulation provide an experimental basis from which such fiber-specific digital twins may be developed [213,214].

(v) Optimization and decision support. The fifth level uses predictive models or estimated digital-twin states to identify improved manufacturing decisions. Machine-learning optimization, Bayesian optimization, reinforcement-learning policies, and model-based strategies can be used to select temperature, extrusion rate, pressure, cooling conditions, fiber tension, or toolpath parameters. When these approaches are evaluated using historical data, simulations, or offline experiments, they should be regarded as process-optimization or decision-support systems rather than as closed-loop controllers. Their transition to real-time control requires sufficiently rapid inference, continuous state updates, explicit physical constraints, uncertainty handling, and reliable communication with the printer or robotic platform. Thus, the key distinction comprises whether the algorithm merely recommends a process setting or whether it continuously modifies the physical manufacturing process in response to measured disturbances.

(vi) Real-time closed-loop and autonomous control. The highest level of manufacturing intelligence is reached when sensing, inference, decision making, and actuation are integrated within the same operational loop. A true closed-loop system therefore requires at least four elements: real-time acquisition of the manufacturing state, online defect diagnosis or state estimation, generation of a corrective action, and experimentally demonstrated execution of that action through the printer or robotic platform. The MPACT-3D framework shown in Figure 10d provides an example of a highly integrated multimodal material-deposition control architecture developed in the broader AM field [215]. Its combination of thermal, visual, vibration, machine-state, physics-guided, and reinforcement-learning control is conceptually relevant to continuous-fiber printing because impregnation and consolidation are likewise governed by coupled thermorheological dynamics. However, MPACT-3D should presently be regarded as a transferable control architecture rather than as a validated 3DP-CFRPC controller. Fiber-specific implementation would require additional state variables—including tow tension, fiber-placement deviation, impregnation state, interfacial temperature, and consolidation quality—and direct closed-loop validation during continuous-fiber deposition [216,217].

LLM-based manufacturing agents represent an even more prospective supervisory layer of manufacturing intelligence. The LLM-3D print concept shown in Figure 10e was developed for general 3D-printing monitoring and control rather than specifically for 3DP-CFRPCs [218]. Such agents may assist in interpreting multimodal observations, linking detected anomalies to candidate process causes, and coordinating high-level corrective decisions [219]. Their transfer to continuous-fiber composite manufacturing, however, remains to be experimentally established. In particular, language-model reasoning should not be coupled directly to unrestricted machine actuation without deterministic safety interlocks, bounded control actions, physics- and process-based constraints, and independent verification of the proposed intervention. LLM-based agents should therefore presently be regarded primarily as supervisory or decision-support technologies for 3DP-CFRPCs rather than as experimentally validated autonomous controllers [220,221].

Within the smart architecture framework, AI-enabled manufacturing should therefore be understood as a hierarchy rather than as a single technology category. Sensing establishes process observability; defect-detection algorithms transform signals into manufacturing information; surrogate models provide prediction; digital twins estimate the evolving physical state; optimization algorithms identify corrective decisions; and closed-loop controllers convert these decisions into physical intervention. Current research regarding 3DP-CFRPCs is comparatively mature at the sensing, defect-detection, and process-monitoring levels, whereas experimentally validated fiber-specific digital twins and fully integrated real-time autonomous control remain substantially less developed. Future progress will therefore depend not only on more sophisticated AI models, but also on reliable multimodal sensing, fiber-specific state variables, synchronized state estimation, uncertainty-aware decision making, low-latency computation, actuator integration, and experimentally validated fail-safe control.

## 6. Mechanical Performance and Reliability of 3DP-CFRPCs

Within the smart architecture framework, the mechanical performance of 3DP-CFRPCs arises from the hierarchical interplay of process-induced microstructures, structural architectures, and loading conditions. Key microstructural features—fiber alignment, interfacial integrity, porosity, and interlayer bonding—dictate load transfer and damage evolution, directly shaping both the instantaneous mechanical response and the long-term structural reliability of printed components. Mechanical characterization, therefore, serves a dual role: it evaluates structural performance while establishing the critical structure–property–reliability linkages that inform feedstock design, process optimization, and intelligent manufacturing.

### 6.1. Microstructure and Its Strengthening Mechanism

**Figure 11:**
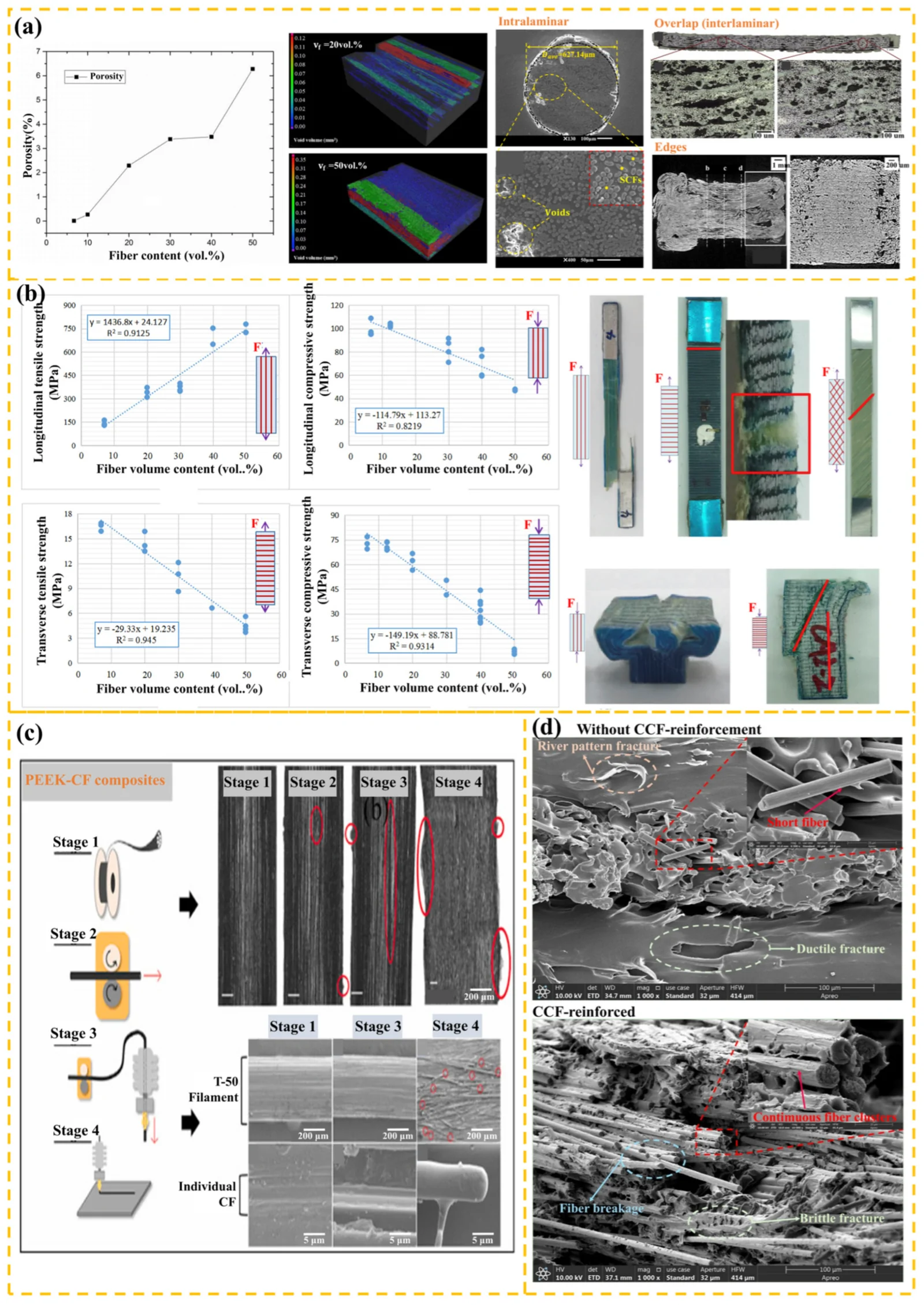
Microstructure, damage evolution, and property variation in 3DP-CFRPCs. (**a**) SEM images of pore distribution and porosity in different regions of the CFRPC [11,16,79,120,121]. (**b**) The strength characteristics and failure modes of CFRPC under different fiber contents and orientations [11]. (**c**) Fiber damage during different printing stages [222,223]. (**d**) Impact-fracture surface morphology of CCF-reinforced and unreinforced specimens [225].

Figure 11 summarizes the multiscale microstructural characteristics commonly observed in 3DP-CFRPCs. At the microscale (Figure 11a), fiber bundles exhibit complex internal architectures consisting of partially impregnated filaments, matrix-rich regions, and heterogeneous interface morphologies. Synchrotron X-ray tomography and high-resolution micro-CT studies revealed that impregnation quality not only determines local stress transfer efficiency but also affects the evolution of crystallinity and residual stress within thermoplastic matrices [11,120,121]. In high-performance CF/PEEK and CF/PAEK systems, localized thermal histories generated during deposition were shown to induce significant variations in crystalline morphology, resulting in spatially dependent stiffness and interlaminar behavior. These findings demonstrate that microstructural heterogeneity extends beyond geometric features and includes thermally induced material-state variations [16,79].

At the mesoscale, 3DP-CFRPCs introduce an additional level of structural complexity through programmable fiber trajectories. As illustrated in Figure 11b, increasing fiber-volume fraction gradually transforms the dominant load-transfer mechanism from matrix-controlled deformation toward fiber-dominated stress carrying. However, structural efficiency is often governed more by fiber continuity and trajectory smoothness than by fiber content alone [11,25]. Curvilinear fiber placement, variable-stiffness architectures, and stress-aligned deposition strategies have been shown to redistribute load paths more effectively than traditional unidirectional layouts, enabling substantial improvements in stiffness, strength, and energy absorption without increasing material consumption [222].

Fiber architecture evolution during manufacturing further contributes to microstructural complexity. Figure 11c highlights how tow morphology continuously changes throughout feeding, impregnation, extrusion, and deposition. The investigations employing in situ imaging and digital image correlation revealed that fiber bundles undergo progressive bending, compaction, spreading, and reorientation during printing [223]. These morphological changes directly influence local fiber packing density, interface area, and load-transfer capability. As a result, the final mechanical performance is determined not only by the deposited geometry but also by the evolution history of the fiber architecture during processing [27,224].

Figure 11d highlights the role of continuous carbon-fiber (CCF) reinforcement in governing the impact-related fracture behavior of printed composites. Compared with the unreinforced sample, the CCF-reinforced specimen exhibits a more heterogeneous but mechanically more effective fracture morphology, characterized by fiber breakage, fiber pull-out, crack bridging, and localized brittle fracture. These features indicate improved fiber–matrix interfacial bonding, more efficient stress transfer, and enhanced crack resistance during loading. In contrast, the unreinforced structure shows predominantly ductile fracture and river-pattern features, reflecting the absence of an effective load-bearing fiber network [225]. From a smart architecture perspective, superior performance increasingly depends on architectural optimization rather than simply increasing reinforcement content. Emerging approaches based on variable fiber-density distributions, graded reinforcement architectures, and topology-guided deposition have shown that strategically tailoring microstructure can achieve comparable or superior mechanical performance with lower material consumption [226,227].

### 6.2. Mechanical Performance and Failure Mechanisms

#### 6.2.1. Interlaminar Shear Strength and Interfacial Failure Mechanisms

**Figure 12:**
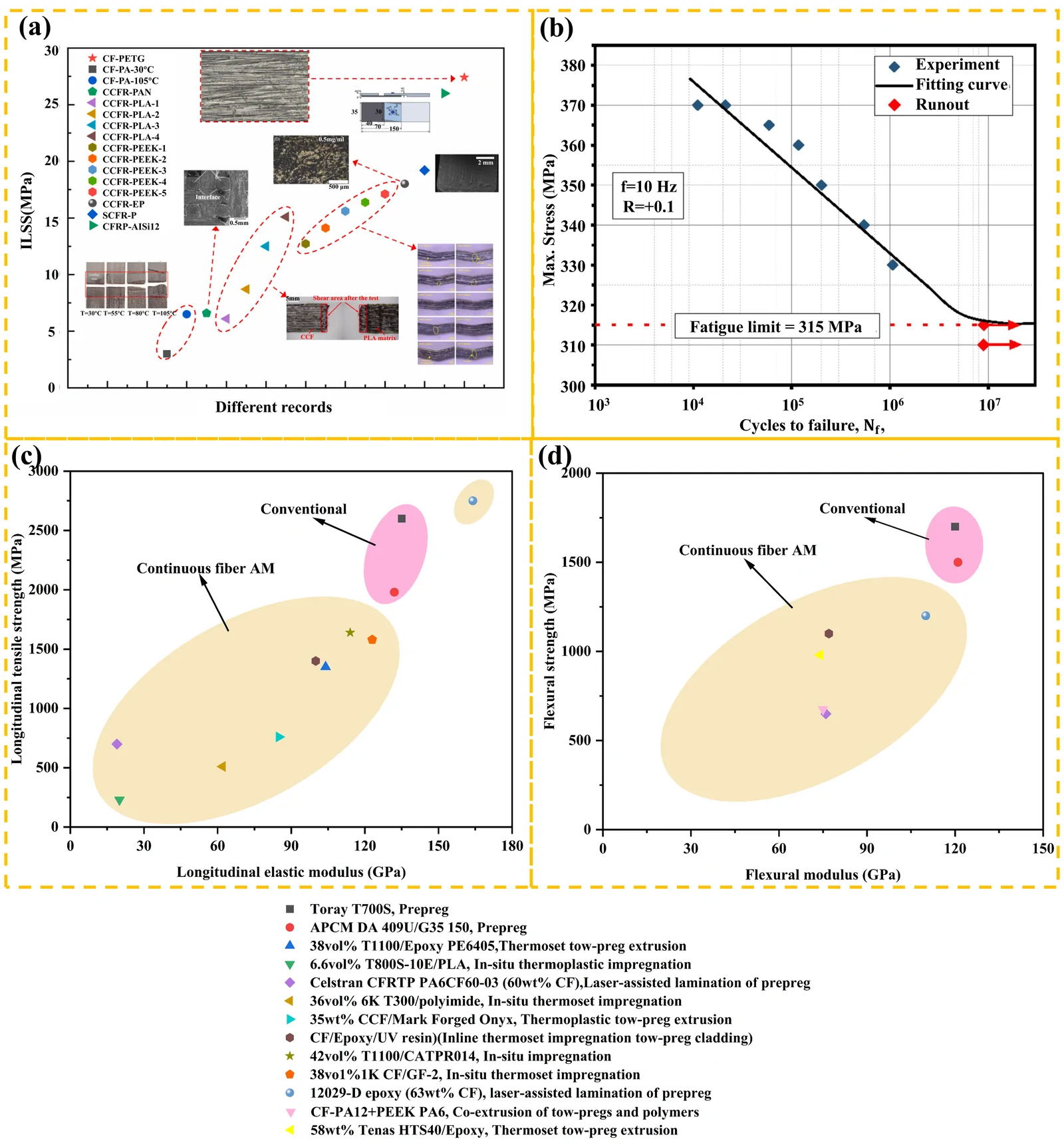
Mechanical properties of 3DP-CFRPCs. (**a**) ILSS [231,232,234,235,237,238,239,241]. (**b**) Experimental stress life (S-N) curve [257]. (**c**) Longitudinal tensile properties [26,73,243,246,248,251]. (**d**) Flexural properties [26,237,243,246,252,254].

Interlaminar shear remains one of the most critical weakness vectors for 3DP-CFRPCs because the layerwise deposition intrinsic to most extrusion-based processes produces comparatively weak interfaces, trapped porosity, and incomplete fiber–matrix wetting. Experimental surveys and reviews consistently report that ILSS of as-printed components is often lower than that of conventionally consolidated laminates, and that measured ILSS is highly sensitive to void content, interfacial adhesion, and local consolidation pressure during deposition [228,229]. As shown in Figure 12a, the interlaminar shear strength (ILSS) of 3DP-CFRPCs exhibits substantial variation among different material systems and manufacturing routes, reflecting the strong dependence of interfacial performance on impregnation quality, consolidation efficiency, and thermal history [230,231,232]. The ILSS values plotted in Figure 12a represent selected material–process combinations rather than the full attainable range of continuous-fiber additive manufacturing. This wide distribution highlights that interfacial integrity remains one of the most critical limitations in 3DP-CFRPCs [233,234,235].

The dominant failure mechanisms are closely associated with the quality of the fiber–matrix interface and interlayer fusion. Inadequate wetting and insufficient polymer diffusion often result in interfacial debonding and crack initiation at the fiber–matrix boundary, whereas improved impregnation and enhanced consolidation promote cohesive failure within the matrix and more effective stress transfer across deposited roads [236,237,238,239]. Therefore, ILSS serves not only as an indicator of interface quality but also as a key predictor of subsequent tensile, flexural, and fatigue performance [240,241].

#### 6.2.2. Longitudinal Tensile Behavior and Failure Mechanisms

Figure 12c compares the longitudinal tensile properties of continuous-fiber AM composites with those of conventionally manufactured laminates. Although significant improvements in tensile modulus and strength have been achieved through optimized fiber placement and impregnation strategies, a noticeable performance gap remains between additively manufactured and aerospace-grade prepreg composites [242,243]. A clear positive correlation can be observed between tensile strength and longitudinal modulus, indicating that reinforcement efficiency is primarily governed by fiber alignment and fiber-volume fraction. Continuous-fiber AM systems employing thermoset impregnation, laser-assisted consolidation, or prepreg-based architectures generally exhibit superior tensile properties due to improved fiber wetting and reduced void content. In contrast, in situ thermoplastic impregnation systems often suffer from incomplete resin infiltration and interfacial imperfections, resulting in lower reinforcement efficiency [244,245,246]. Failure under longitudinal tension is typically characterized by a combination of fiber fracture, interfacial debonding, matrix cracking, and fiber pull-out. The relative contribution of these mechanisms depends strongly on impregnation quality and defect distribution. Well-consolidated composites tend to exhibit fiber-dominated failure, whereas poorly impregnated structures often fail prematurely through interface-controlled damage evolution [247,248].

#### 6.2.3. Flexural Performance and Structural Load-Bearing Capacity

Flexural behavior provides a more comprehensive assessment of structural performance because it simultaneously involves tensile, compressive, and interlaminar stress states [26,73,249]. As illustrated in Figure 12d, continuous-fiber AM composites demonstrate significant improvements in flexural strength and modulus compared with the results for unreinforced thermoplastics, although their performance remains sensitive to process-induced defects and fiber architecture [250]. The observed variation in flexural properties is primarily associated with differences in fiber continuity, path planning, impregnation uniformity, and interlayer bonding quality. Since bending loads generate tensile stresses on one side of the structure and compressive stresses on the opposite side, local defects such as fiber waviness, void clusters, and weak interfaces can trigger premature failure through matrix cracking, fiber buckling, or delamination [251,252]. These observations indicate that flexural performance is not solely determined by constituent materials but also by the effectiveness of stress redistribution within the printed architecture. Consequently, topology optimization, continuous-path planning, and improved consolidation strategies are becoming increasingly important for maximizing the structural efficiency of 3DP-CFRPCs [253,254].

#### 6.2.4. Fatigue Behavior and Durability Evolution

Fatigue characterization of 3DP-CFRPCs is an emerging but rapidly expanding area. The experimental work (covering rotating–bending, tension–tension, and tension–compression regimes) shows that the introduction of continuous fibers dramatically improves fatigue endurance relative to that of unreinforced printed polymers, yet absolute fatigue life is still strongly modulated by printing defects, fiber continuity, and matrix toughness [255]. Typical fatigue damage progression begins with matrix microcracking around fiber bundles and voids, followed by fiber–matrix debonding and progressive fiber breakage; when delamination paths exist, they often provide low-energy routes for accelerated fatigue failure [256]. Figure 12b demonstrates that fatigue in 3DP-CFRPCs is an emergent, process-dependent system response, not a single material constant, where process-induced microdefects dominate scatter and crack initiation. Thus, robust fatigue design requires probabilistic descriptors, in situ damage monitoring, and multi-scale progressive-damage models to mechanistically link defect evolution to macroscopic life predictions for safety-critical applications [2,257].

Parametric studies indicate that layer thickness, nozzle/bed temperature profile, printing speed, fiber-volume fraction, and raster orientation all influence fatigue endurance. Optimization studies using design-of-experiments and Taguchi methods have been applied to short-fiber FDM systems and are now being extended to continuous-fiber processes; these show that manufacturing parameters that reduce porosity and improve consolidation (e.g., finer layer heights, slower speeds, elevated bed/nozzle temperatures) generally increase fatigue life, but tradeoffs with throughput and thermal degradation must be carefully managed [258,259].

Because full S–N characterization is time consuming, researchers are increasingly combining accelerated fatigue testing with multiscale numerical models to predict residual stiffness/strength evolution and service life [91,260]. Multiscale and progressive damage models explicitly include layerwise defect statistics (void distribution, imperfect impregnation) and interfacial damage laws; when calibrated with targeted experiments, these models can capture the observed stiffness decay and the transition from matrix-dominated to fiber-dominated failure regimes under cyclic loading. This combined experimental–computational paradigm aligns with the smart architecture principle stating that process, defect, and performance must be treated as a coupled system rather than as independent variables. This approach can support the design of more reliable printing strategies for fatigue-critical applications [261,262].

### 6.3. Minimum Reporting and Benchmarking Requirements for Mechanical Qualification

The large scatter in reported tensile, flexural, interlaminar, and fatigue properties of 3DP-CFRPCs demonstrates that mechanical values cannot be interpreted independently of the corresponding as-manufactured state. Meaningful cross-study comparison, therefore, requires a minimum reporting framework that simultaneously documents material, process, architecture, test configuration, and statistical uncertainty. At the material level, studies should report the fiber/matrix system, reinforcement form, nominal and measured fiber-volume fraction, void content, measurement method, and, where relevant, the crystallinity or cure state. Manufacturing metadata should include the impregnation/deposition route, printer architecture, fiber-path strategy, build direction, local fiber orientation, printing speed, layer thickness, thermal and consolidation conditions, and post-processing history, with printing direction explicitly defined relative to both specimen geometry and loading direction. Mechanical testing should additionally report specimen dimensions, gauge length or support span, conditioning and environmental conditions, applicable standard and any deviations from it, loading rate, and dominant stress state. For fatigue characterization, loading mode, stress ratio, waveform, frequency, stress amplitude or normalized stress level, specimen number, failure definition, and run-out criterion should be provided. Reported properties should include the number of independent specimens, together with mean values and standard deviation or coefficient of variation, because scatter in 3DP-CFRPCs reflects not only experimental uncertainty but also manufacturing repeatability. The purpose of such reporting is not to impose a universal performance threshold, but to establish the metadata required for traceable benchmarking, stratified cross-study comparison, interlaboratory validation, process qualification, and ultimately, application-specific certification.

## 7. Conclusions and Outlook

This review establishes smart architecture as an integrated framework for understanding extrusion-based 3DP-CFRPCs through the coupled evolution of feedstock characteristics, process physics, fiber architecture, defects, multifunctionality, and manufacturing intelligence. The central conclusion is that future progress will depend less on maximizing isolated material or process parameters than on achieving quantitative, reproducible, and traceable control of the complete process–state–architecture–performance chain.

At the materials and process level, impregnation and interfacial reliability remain the principal bottlenecks. Matrix rheology, fiber-bundle permeability, temperature, pressure, deposition speed, and fiber tension jointly govern wetting, porosity, fiber-volume fraction, interlayer bonding, and residual stress. Industrial scale-up, therefore, requires not only broader processing windows, but also reduced batch-to-batch and machine-to-machine variability, stable tow handling, controlled tool condition, and repeatable consolidation under increasing deposition rates. The relevant objective is not a single high-performance specimen, but reproducible microstructural quality across machines, builds, and production batches.

At the architecture and structural-reliability level, topology optimization and fiber-path planning must move from idealized design toward manufacturability-, defect-, and uncertainty-aware synthesis. Minimum steering radius, tow width, fiber continuity, tension, cutting/restart, local overlap, collision avoidance, and multi-axis kinematics should be incorporated directly into optimization and verified against the as-manufactured architecture. At the same time, structural qualification remains limited by variability in porosity, fiber placement, interfaces, and test procedures. Standardized defect characterization, statistically meaningful material allowables, fatigue and environmental-durability assessment, inspection protocols, repair criteria, and certification-oriented component testing are therefore essential, particularly for aerospace and other safety-critical applications.

Multifunctionality and intelligent manufacturing represent important opportunities, but both require more rigorous validation. Self-sensing, self-healing, morphing, thermal management, and embedded electronics should be evaluated using coupled structural–functional metrics so that added functionality does not compromise fiber continuity, interfacial integrity, fatigue resistance, or long-term durability. Likewise, AI-enabled manufacturing should progress through a clear hierarchy, from sensing and defect detection to state estimation, optimization, and experimentally validated closed-loop control. Digital twins and autonomous agents will become valuable only when they are linked to reliable sensing, uncertainty-aware models, bounded corrective actions, and demonstrable improvements in manufacturing repeatability.

Ultimately, the industrial transition of 3DP-CFRPCs is governed by three interconnected barriers: material-state consistency, manufacturing-state control, and qualification/economic viability. Autonomous control can reduce process variability, but it cannot substitute for durability validation, certification, repairability, end-of-life strategies, or productivity assessment relative to automated fiber placement and conventional composite manufacturing. The key metric for future development should therefore shift from peak coupon-level properties toward the reproducible manufacturing of defect-tolerant, structurally efficient, multifunctional, and qualifiable composite architectures with traceable quality and competitive lifecycle performance. In this context, smart architecture provides a pathway for integrating materials, processing, design, performance assessment, and digital intelligence into a closed-loop and industrially relevant composite-manufacturing framework.

## Figures and Tables

**Table 1:** Comparison of representative recent reviews on continuous-fiber additive manufacturing and the positioning of the present review.

Representative Review	Principal Scope	Main Contribution	Positioning Relative to the Present Review
Wong et al. (2023) [29]	Fiber-reinforced polymer AM; design methodologies	Multiscale topology optimization, fiber-orientation parameterization, micromechanics, process planning	Strong design/manufacturing perspective; smart architecture further connects process-induced interface/defect states with multifunctionality, reliability and closed-loop control
Cheng et al. (2023) [38]	3D/4D printed functional CFRPCs	Functional materials, shape memory/morphing, self-monitoring and self-healing	Focused on multifunctional/4D behavior; present review treats functionality as one output of coupled feedstock–process–architecture evolution
Karimi et al. (2024) [49]	FDM mechanisms for fiber-reinforced composites	In situ and ex situ/prepreg manufacturing mechanisms and reinforcement routes	Process-centered comparison; present review additionally connects impregnation/consolidation to defect states, architecture and reliability
Jamal et al. (2024) [50]	FDM of continuous-fiber CFRPCs	Printing parameters and their effects on mechanical properties	Primarily parameter–property oriented; present review emphasizes intermediate interface/defect states and cross-domain feedback
Zheng et al. (2025) [17]	Materials, processing, mechanics and functional applications	Broad integration of material selection, process, mechanical-property regulation and functional applications	Closest broad-scope review; smart architecture differs primarily by organizing these topics through explicit coupling/state variables and a bidirectional manufacturing-control loop
Zhang et al. (2025) [10]	Continuous carbon-fiber thermoplastic AM	Materials, processes, performance enhancement, interface/void defects and failure	Detailed CFRTP performance/failure synthesis; present review spans wider architectures and treats defects as intermediate states linking process, design and intelligent control
Cheng et al. (2025) [46]	Non-planar continuous-fiber printing	Non-planar processes, materials, multi-axis manufacturing and process challenges	Focused on non-planar manufacturing; present review embeds multi-axis/non-planar printing within a broader material–process–architecture–performance framework
Zhao et al. (2025) [47]	Process planning and intelligent control	Slicing, path planning, manufacturing constraints, AI-assisted monitoring/control	Strong planning/control focus; present review connects these control strategies upstream to feedstock/interface evolution and downstream to multifunctionality and structural reliability
Nozawa and Serhat (2025) [51]	Topology and fiber-path optimization	Sequential/concurrent topology–fiber optimization, robustness, manufacturability and experimental verification	Strong structural-design focus; present review treats optimization as one layer within a closed-loop process–defect–architecture–performance system

## Data Availability

No new data were created or analyzed in this study. Data sharing is not applicable to this article.

## Abbreviations

The following abbreviations are used in this manuscript:

Abbreviation	Full name
AI	Artificial Intelligence
AM	Additive Manufacturing
ANDFO	Adaptive Normal Distribution Fiber Optimization
AFP	Automated Fiber Placement
BESO	Bi-Directional Evolutionary Structural Optimization
CCF	Continuous Carbon Fiber
CFRPC	Continuous-Fiber-Reinforced Polymer Composite
DGTO	Derivable Geodesics-Coupled Topology Optimization
DoF	Degree of Freedom
FDM	Fused Deposition Modeling
FFF	Fused Filament Fabrication
HDI	Hybrid-Dispersion Impregnation
ILSS	Interlaminar Shear Strength
ISTO	Integrated Structure and Toolpath Optimization
LCE	Liquid-Crystal Elastomer
LDED	Laser Direct Energy Deposition
LLM	Large Language Model
MDD	Minimum Detectable Damage
MEX	Material Extrusion
MMC	Moving Morphable Components
MPACT-3D	Multimodal Physics-Aware Autonomous Control for 3D Printing
PA	Polyamide
PAEK	Polyaryletherketone
PEEK	Polyether ether ketone
PEI	Polyetherimide
PLA	Polylactic Acid
PPS	Polyphenylene Sulfide
RAAM	Robotic Additive Manufacturing
SOMP	Solid Orthotropic Material with Penalization
SMPC	Shape-Memory Polymer Composites
S–N	Stress–Number of Cycles
TO	Topology Optimization
UV	Ultraviolet
YOLO	You Only Look Once
3DP-CFRPCs	3D-Printed Continuous-Fiber-Reinforced Polymer Composites
